# Cellular TRIM33 restrains HIV-1 infection by targeting viral integrase for proteasomal degradation

**DOI:** 10.1038/s41467-019-08810-0

**Published:** 2019-02-25

**Authors:** Hashim Ali, Miguel Mano, Luca Braga, Asma Naseem, Bruna Marini, Diem My Vu, Chiara Collesi, Germana Meroni, Marina Lusic, Mauro Giacca

**Affiliations:** 1 Molecular Medicine Laboratory, International Centre for Genetic Engineering and Biotechnology (ICGEB), Padriciano 99, 34149 Trieste, Italy; 2 Department of Cardiovascular Medicine & Sciences, King’s College London, The James Black Centre, 125 Coldharbour Lane, London, SE5 9N UK; 3 Cellular Immunology Laboratory, International Centre for Genetic Engineering and Biotechnology (ICGEB), Padriciano 99, 34149 Trieste, Italy; 4 Department of Medical, Surgical and Health Sciences, University of Trieste, 34127 Trieste, Italy; 5 Department of Life Sciences, University of Trieste, 34127 Trieste, Italy; 6 Present Address: Center for Neuroscience and Cell Biology (CNC), University of Coimbra, Coimbra, 3060-197 Portugal; 7 Present Address: Ulisse BioMed S.r.l., AREA Science Park, Basovizza, 34149 Trieste, Italy; 8 Present Address: University Hospital Heidelberg and German Center for Infection Research, 69120 Heidelberg, Germany

## Abstract

Productive HIV-1 replication requires viral integrase (IN), which catalyzes integration of the viral genome into the host cell DNA. IN, however, is short lived and is rapidly degraded by the host ubiquitin-proteasome system. To identify the cellular factors responsible for HIV-1 IN degradation, we performed a targeted RNAi screen using a library of siRNAs against all components of the ubiquitin-conjugation machinery using high-content microscopy. Here we report that the E3 RING ligase TRIM33 is a major determinant of HIV-1 IN stability. CD4-positive cells with TRIM33 knock down show increased HIV-1 replication and proviral DNA formation, while those overexpressing the factor display opposite effects. Knock down of TRIM33 reverts the phenotype of an HIV-1 molecular clone carrying substitution of IN serine 57 to alanine, a mutation known to impair viral DNA integration. Thus, TRIM33 acts as a cellular factor restricting HIV-1 infection by preventing provirus formation.

## Abstract (web summary)

HIV-1 integration into host DNA is mediated by the viral integrase (IN). Here, using siRNA screen and high-content microscopy, the authors identify the host E3 RING ligase TRIM33 to affect IN stability and show that TRIM33 prevents viral integration by triggering IN proteasome-mediated degradation.

## Introduction

Integration into the host cell genome, which is catalyzed by the virus-encoded integrase (IN) enzyme, is a hallmark of all members of the Retroviridae family^1,2^. In both lenti- and gamma- retroviruses, functionally active IN is a product of endo-proteolytic cleavage of the Gag-Pol polyprotein by action of the virally encoded protease. As a result of this process, in the case of HIV-1, mature IN harbors an N-terminal phenylalanine, which renders the protein susceptible to rapid degradation by the 26S proteasome following recognition by the class of E3 ubiquitin ligases known as recognins (N-end rule ubiquitin E3 ligases), which recognize N-degron signals^3,4^. When the first amino-acid of HIV-1 IN is mutated to methionine, IN stability increases, however the protein is still short-lived^4–8^, an indication that IN is targeted for degradation through the proteasomal pathway also independent from N-terminal recognition. Indeed, this conclusion is consistent with the long-standing observation that inhibition of the proteasome enhances HIV-1 infection^9,10^.

The 160-kDa HIV-1 Gag-Pol polyprotein is packaged into virions preceding proteolytic processing, which occurs in the virions after budding. Upon target cell infection, mature IN (32 kDa) is part of the viral pre-integration complex (PIC), which provides a secluded environment where reverse transcription of viral RNA into blunt-ended, linear DNA takes place^11^. Part of the PIC is then transported into the nucleus, where viral IN eventually exerts its enzymatic function. Here, the protein enters in contact with various nuclear proteins, including factors that increase its efficacy and protect it against proteasomal degradation. These include the transcriptional coactivator lens epithelium-derived growth factor/transcription coactivator p75 (LEDGF/p75)^5,12,13^ and Ku70, a component of the cellular double-stranded DNA break repair through the non-homologous end-joining pathway^14^. For both factors, binding to IN was shown to prevent its proteasomal degradation^7,14^.

In addition, our previous work has shown that IN stability, and thus enzymatic function, is increased by post-translational modification. Phosphorylation of serine 57 (S57) in the IN catalytic core by cellular c-Jun N-terminal kinase (JNK) renders the protein a substrate for cis/trans isomerization by the peptidyl-prolyl isomerase Pin1; this induced structural modification markedly increases IN half-life by reducing its ubiquitination and is required for efficient HIV-1 infection^15^. A point mutation in IN(S57) leads to accelerated IN degradation and severely restricts infectivity of the virus. Consistent with the stabilizing role of JNK-induced IN(S57) phosphorylation, lack of JNK expression restricts viral infection in resting, primary CD4+ T lymphocytes^15^.

Taken together, these studies indicate that, in the infected cells, IN is a substrate for degradation by the ubiquitin-proteasomal pathway. This pathway consists in the sequential action of three different classes of enzymes. The 76 aa-polypeptide ubiquitin is first activated by binding to one of a few E1 ubiquitin-activating enzymes, to be then transferred to one of ~40 E2 conjugation enzymes, which act in conjunction with over 600 E3 ubiquitin protein ligases, which provide target specificity by recognizing the proteins to be tagged and eventually transferring ubiquitin to them^16–19^. The poly-ubiquitinated substrate proteins are then recognized by the 26S proteasome machinery and degraded into short peptides^20^. E3 ligases are classified into two main classes (RING and HECT) based on conserved structural domains and the molecular mechanism of ubiquitin transfer to the substrate. The RING (really interesting new gene)-type E3 ligases catalyze direct transfer of ubiquitin from the ubiquitin-loaded E2 enzyme to the substrate, concurrently binding with the cognate E2 and the substrate^17,21^. In contrast, the HECT (homology to E6AP C-terminus)-type E3 ligases require two steps to transfer ubiquitin to the substrate, with ubiquitin being first transferred from the E2 to an active site cysteine in the E3 and then from the E3 to the substrate^22,23^.

As a consequence of this mechanism, it can be predicted that, in HIV-1 susceptible cells, one or more cellular E3 ligases must exist, in addition to those involved in N-terminal recognition, which target IN for degradation through the ubiquitin-proteasome pathway, thus hampering HIV-1 infection by impairing viral cDNA integration. The identity of these E3 ligases, however, has escaped identification so far.

Here we report the results of a high-throughput screening (HTS) using a library of siRNAs against the whole set of components of the ubiquitin-conjugation machinery, including E1 and E2 enzymes and E3 RING and HECT ligases (598 target genes) using high-content microscopy, in search for factors that drive IN ubiquitination and degradation. We report that the cellular E3 RING ligase TRIM33 is a major determinant of HIV-1 IN stability inside the cells binding the IN carboxy-terminal domain through its RING portion and determining its poly-ubiquitination. Lack of TRIM33 overcomes the effect of Pin1 knock-down and rescues infectivity of an HIV-1 mutant carrying the destabilizing IN (S57) mutation. Thus, TRIM33 acts as an inhibitory factor hampering HIV-1 infection by decreasing IN function and thus preventing viral cDNA integration into the host cell genome.

## Results

### Cellular ubiquitin-proteasome targets HIV-1 IN for degradation

**Fig. 1:**
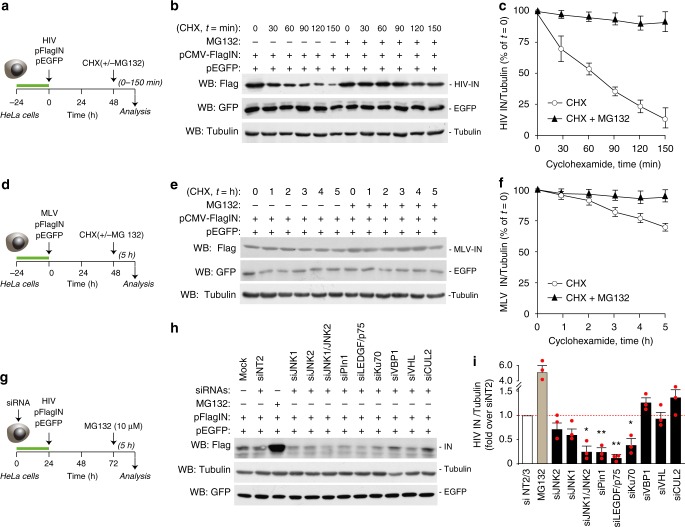
Degradation of retroviral integrases by the host ubiquitin-proteasome system. **a** Schematic representation of the HIV-1 IN stability assay. HeLa cells were cotransfected with plasmids expressing Flag-tagged IN or EGFP; 48 h later, cells were treated with cycloheximide (CHX) in the presence and absence of the proteasome inhibitor MG132. **b** Measurement of HIV-1 IN stability. Flag-IN-expressing HeLa cells were treated with CHX (30 μg/ml) with or without MG132, followed by protein detection at different time points using an anti-Flag antibody. Tubulin levels served as a loading control and EGFP levels as a transfection and specificity control. **c** Quantification of HIV-1 IN protein levels in the presence and absence of MG132 in CHX-treated cells at the indicated time points. Data are mean ± SEM; *n* = 3 independent experiments. **d** Schematic representation of the MLV IN stability assay. **e** Measurement of MLV- IN stability. Experiments were performed as in panel **c**. **f** Quantification of MLV-IN protein levels. Data are mean ± SEM; *n* = 3 independent experiments. **g** Knock-down of cellular factors to assess HIV-1 IN levels. HeLa cells were transfected with a plasmid expressing pFlag-IN and either transfected 24 h in advance with siRNAs against selected cellular factors or treated, 48 h later, with MG132. **h** Representative experiment showing the levels of HIV-1 IN after knock-down of selected cellular factors by RNAi, as indicated. Cell treatment with MG132 served as a positive control. IN levels were analyzed in whole cell lysates by immunoblotting with anti-Flag antibody. Immunoblotting for Tubulin served as a loading control. The bottom panel shows a western blotting for EGFP in a representative experiment to show lack of effect of any of the tested siRNAs on EGFP levels. **i** Quantification of HIV-1 IN levels in whole cell extracts of cells in which the indicated factors were knocked down by siRNA transfection. Data are normalized for Tubulin levels and expressed as fold over cell treatment with non-targeting NT2 siRNA control. Data are mean ± SEM; *n* = 3 independent experiments; *P < 0.05; **P < 0.01; one-way ANOVA

We examined HIV-1 IN protein stability in HeLa cells upon transfection of a plasmid expressing Flag-tagged IN^15^ in the presence or absence of the proteasome inhibitor MG132 in cells treated with cycloheximide (CHX, 30 μg/ml), which blocks de novo protein synthesis. The levels of IN progressively diminished over time, with an estimated half-life of ~60 min, in line with our previous results^15^; proteasome inhibition maintained high protein levels over time (Fig. 1a for experimental scheme, Fig. 1b representative immunoblot and Fig. 1c for quantification of three independent experiments). The levels of EGFP expressed from a separate plasmid transfected together with IN in the same cells were unaffected by the treatments and remained stable over time (Fig. 1b). In similar experiments, the half-life of IN from the Moloney-Murine Leukemia Virus (MLV) gammaretrovirus was > 5 h and its sensitivity to proteasomal degradation was minimal (Fig. 1e, f). Collectively, these results indicate that HIV-1 IN, when expressed inside the cells, is subject to relatively rapid proteasome-mediated degradation.

We asked whether silencing of some of the established interacting partners of HIV-1 IN might affect its levels. We found that siRNAs against LEDGF/p75, Ku70, Pin1, and the combination of JNK1 and JNK2 significantly reduced the amount of IN (Fig. 1g–i for experimental scheme, representative immunoblot, and quantification, respectively). We also knocked down the VBP1 protein, which was reported to tether IN to the von Hippel Lindau (VHL)/Cullin 2 (CUL2) ubiquitin degradation complex, and VHL and CUL2 themselves; in none of these cases, however, IN levels were increased. For all these knock-downs, immunoblotting analysis revealed >65% downregulation of the respective siRNA targets (Supplementary Fig. 1). None of the siRNAs had an effect on the levels of the EGFP protein expressed in the same cells in a separate experiment (Fig. 1h).

Collectively, these results indicate that the host ubiquitin-proteasome system targets HIV-1 IN for degradation through a still unrecognized ubiquitin ligase. They also show that a few cellular factors protect IN from degradation.

### High throughput screening for cellular factors responsible for HIV-1 IN levels

**Fig. 2:**
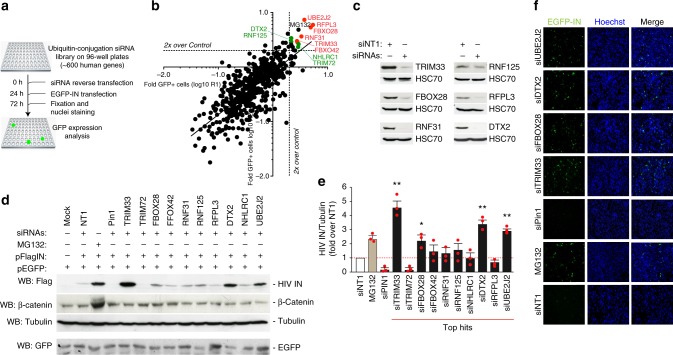
High-throughput, siRNA-based screening to identify cellular factors regulating HIV-1 integrase stability. **a** Workflow for the siRNA-based screening. Cellular fluorescence, as surrogate of IN levels, was analyzed by automated, high-content fluorescent microscopy. **b** Results of screening. The graphs show the log10 values of the fold change of EGFP-positive cells over control in the two replicate screenings (R1 and R2). The dotted lines show 2x increase over Control (pool of results using 4 non-targeting siRNAs and mock-transfected cells). The 6 siRNAs in red are those that were in the top 10 in both screenings. The 4 siRNAs in green are those that were among the top 10 in one of screening while anyhow showing an effect ≥2 fold over control in the other screening. The effect of MG132 is shown in blue. **c** Confirmation of effective silencing of Pin1, TRIM33, FBOX28, RNF31, RNF125, RFPl3, and DTX by immunoblotting with the respective antibodies. Cells transfection of non-targeting siRNA1 (siNT1) was used as a control. HSC70 served as a loading control. **d** Representative immunoblot showing the levels of HIV-IN after knock-down of the top 10 E3 ligases from the screening. HeLa cells were transfected with siRNAs against the identified factors or with a siRNA against Pin1, followed by transfection of Flag-IN and EGFP. Forty-eight hour later the levels of IN were assessed by anti-Flag immunoblotting. Tubulin served as a loading control; β-catenin was used to confirm effect of the MG132 treatment. The bottom panel shows a western blotting for EGFP in a representative experiment to show lack of effect of any of the tested siRNAs on EGFP levels. **e** Quantification of the levels of HIV-1 IN after knock-down of the top 10 E3 ligases identified by the screening. Experiments were performed as in panel **d**. IN levels are expressed after normalization for tubulin and as fold over siNT1. Data are mean ± SEM; *n* = 3 independent experiments; *P < 0.05; **P < 0.01; one-way ANOVA. **f** Representative high-content microscopy images showing of EGFP-IN-expressing cells after depletion of four cellular ubiquitin-conjugation factors (TRIM33, FBXO28, siDTX2, and siUBE2J2) or Pin1

To identify host factors responsible for IN degradation, we performed a high-content, fluorescence microscopy-based, high throughput screening (HTS) using a library of siRNAs against factors in the ubiquitin-conjugation system, including E1 and E2 enzymes and E3 RING and HECT domain ligases (598 target genes, 4 siRNAs per target, pooled). HeLa cells were reverse-transfected in 96-well plates with each siRNA pool (efficiency of transfection >85%), in duplicate, and 24 h later were transfected with EGFP-IN. After further 48 h, the cells were fixed, nuclei were counterstained with Hoechst 33342 and GFP fluorescence was analyzed by high-content microscopy (workflow shown in Fig. 2a; 2500 cells in the duplicates were analyzed per experimental condition). The EGFP-moiety did not interfere with IN degradation, since the fusion protein responded to proteasome inhibition in a dose-responsive manner similar to the wild-type IN (Supplementary Fig. 2a, b). In cells expressing the protein and treated with CHX and MG132, the levels of EGFP-IN progressively decreased over time, with an estimated half-life of ~60 min (Supplementary Fig. 2c, d, similar to Flag-tagged IN. Cell viability in the screening was assessed by counting nuclei number; after assessing normal distribution (Shapiro-Wilk test), siRNAs exerting a toxic effect Zscore *P* ≤ 0.10) were excluded from further analysis.

Two independent replicates of the screening were conducted; the replicates showed good reproducibility (Spearman *r* = 0.74; Fig. 2b). Results of the two screening for the 557 siRNAs not impairing cell viability, expressed as log10-fold over mock control, are reported in Supplementary Table 1. Cell treatment with MG132 and with a non-targeting (NT) siRNA (both performed in quadruplicate) served as internal controls.

These replicate screenings identified 35 siRNAs that increased the percentage of EGFP fluorescent cells more than twofold than control. We focused our attention on the 6 genes the knock-down of which was among the top 10 most effective in both screenings (in red in Fig. 2b and Supplementary Table 1) and on the 4 additional genes the knock down of which was among the top 10 in one of screening while anyhow showing an effect ≥2 fold over control in the other screening (in green in Fig. 2b and Supplementary Table 1).

These included the E2 protein UBE2J2 (Ubc6), an ER-associated E2 conjugating enzyme involved in the degradation of misfolded cellular proteins^24,25^ and nine E3 ubiquitin ligases. Two of these were F-box proteins, which form functional complexes with SKP1 and CUL to generate multisubunit E3 ligases (SCF)^26^: FBXO42 and FBXO28, the latter known to be an important regulator of cell cycle progression^27,28^. Other E3 ligases were RFPL3, shown to increase HIV-1 integration by modulating the activity of the HIV-1 PICs^29^; RNF31 (also known as HOIP), a component of the linear ubiquitin chain assembly complex (LUBAC), an E3 ligase that generates linear poly-ubiquitin chains^30^; DTX2, a member of deltex protein family, acting as a positive regulator of Notch signaling^31,32^; NHLRC1/Malin, essential for the ubiquitin-mediated degradation of Laforin^33^, mutations of which lead to Lafora disease^34,35^; RNF125 (also known as TRAC-1, which positively regulates T-cell activation^36^ - this protein is known to downregulate HIV-1replication at the transcriptional level in a ubiquitin–proteasome dependent manner^37^ and is a component of the innate immune response^38^–; and, finally, two members of the TRIM family, which harbors antiviral activities against RNA and DNA viruses^39,40^, TRIM72 (also known as mitsugumin-53, MG53), a skeletal and cardiac tissue-specific protein involved in the plasma membrane repair^41^ and TRIM33, alias TIF1γ/RFG7/PTC7/Ectodermin, which belongs to the Transcriptional Intermediary Factor1 (TIF1) subfamily of TRIM proteins^42^ and has variously been implicated in tumorigenesis^43^.

We wanted to ensure specificity of the HTS results for these top 10 hits. First, we tested the effects of each of the siRNA pools by immunoblotting lysates of siRNA-treated cells with antibodies against the respective proteins. The levels of 6 of the investigated proteins (TRIM33, FBOX28, RNF31, RNF125, RFPL3, and DTX2) were effectively decreased after siRNA treatment (>85% decrease; Fig. 2c), while none of the commercially available antibodies we could test were active against TRIM72, FBOX42, NHLRC1, and UBE2J2. Second, we tested the top 10 siRNA pools to ensure that they effectively upregulated the levels of IN by immunobloting of cell lysates expressing IN-Flag. We found that four siRNAs (against TRIM33, FBOX28, DTX2, and UBE2J2) increased IN protein levels significantly; β-catenin, which is actively degraded by the ubiquitin proteasome pathway^44^, served as an endogenous control in these experiments (representative blot in Fig. 2d and quantification in Fig. 2e). The effect of these four siRNA pools in cells expressing EGFP-IN is shown in Fig. 2f. Those against TRIM33, FBOX28, and DTX2 were specific for IN, since they did not affect the levels of EGFP alone transfected together with IN (separate experiment at the bottom Fig. 2d). Of interest, treatment with the same siRNA pools also upregulated IN levels in cells in which Pin1 had been downregulated, thus determining IN instability^15^ (Supplementary Fig. 2e, f for a representative immunoblot and quantification, respectively). In particular, the knock-down of TRIM33 in Pin1-depleted cells restored IN at levels comparable to those determined by MG132, consistent with the conclusion that IN instability induced by Pin1 knockdown is mediated by TRIM33-mediated IN degradation. Third, we deconvoluted the four siRNA pools against TRIM33, FBOX28, DTX2, and UBE2J2 by individually testing the effect, on the respective protein level and on IN stability, of the four individual siRNAs composing each of these pools. Three out of four siRNAs against TRIM33 decreased the levels of this protein and, at the same time, increased those of IN (Supplementary Fig. 2g). In the cases of the FBOX28, DTX2, and UBE2J2 siRNAs, instead, there was inconsistency between the extent of the respective protein knock-down and the increase in IN levels (Supplementary Fig. 2h–j). Considering these findings, and after having observed the marked increase in IN levels after TRIM33 knock-down, we decided to concentrate our attention on this protein in the subsequent experiments.

### TRIM33 is the only TIF1 family member that regulates HIV-1 integrase stability

**Fig. 3:**
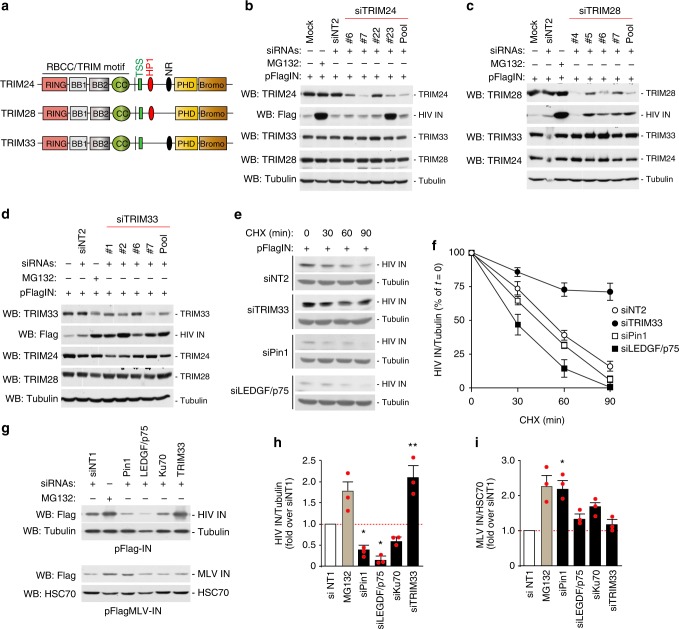
Cellular TRIM33 is only TIF1 family member that negatively regulates HIV-1 integrase stability. **a** Schematic representation of domain organization of TIF1 subfamily members. **b** Effect of anti-TRIM24 siRNAs. The immunoblots show the levels of TRIM24, TRIM33, TRIM28, and HIV-1 IN in HeLa cells transfected with individual siRNAs against TRIM24 (#4, #5, #6, and #7), their pool (Pool) or non-targeting siRNA-2 (siNT2). MG132 was used to assess the effect of inhibiting proteasome-mediated protein degradation. **c** Effect of anti-TRIM28 siRNAs. Same as in panel **b** using siRNAs against TRIM28. **d** Effect of anti-TRIM33 siRNAs. Same as in panel **b** using siRNAs against TRIM33. **e** Stability of HIV-1 IN protein in siRNA-treated cells. Representative immunoblots showing the levels of HIV-1 IN after HeLa cell transfection with siRNAs against TRIM33, Pin1, and LEGF/p75 at different times and treatment with cycloheximide (30 µg/ml) to block protein degradation. Tubulin served as a loading control. **f** Quantification of HIV-1 IN levels. Experiments were performed as in panel **e**. IN amounts are normalized to Tubulin and expressed as percent over IN at time = 0 for each treatment. Data are mean ± SEM; *n* = 3 independent experiments. **g** Effects of siRNAs on the levels of HIV-1 and MLV IN proteins. The figure shows representative immunoblots using lysates of HeLa cells transfected to express either Flag-IN from HIV-1 (upper part) or MLV-IN (lower part)and siRNAs against the indicated factors. Tubulin and HSC70 served as loading controls. **h** Quantification of the effects of siRNAs against the indicated factors or treatment with MG132 on the levels of HIV-1 IN. IN levels are expressed after normalization for tubulin and as fold over non-targeting siRNA siNT1. Data are mean ± SEM; *n* = 3 independent experiments; *P < 0.05; **P < 0.01; one-way ANOVA. **i** Same as in panel **g** for MLV-IN. Normalization was for cellular HSC70. Data are mean ± SEM; *n* = 3 independent experiments; *P < 0.05; one-way ANOVA

The TIF1 subfamily of TRIM proteins contains four members: TRIM24, TRIM28, TRIM33, and TRIM66. The first three TIF1 proteins are highly conserved in domain organization and amino acid sequence (Fig. 3a) and were shown to form complexes in hepatocellular carcinoma cells^45,46^. Therefore, we asked whether other TRIM33 family members also affected IN stability. We silenced TRIM24 and TRIM28 using both siRNA pools and individual siRNAs against the two proteins – obtained after pool deconvolution –, in cells expressing HIV-1 IN. In neither case we observed increased IN levels despite effective protein knock-down; cell treatment with MG132 served as a control for these experiments (Fig. 3b, c for TRIM24 and TRIM28, respectively). An increase in IN levels was only observed in cells transfected with siRNA #23 against TRIM24, which we attribute to an off-target, unspecific effect. In these experiments, we also checked whether the specific knockdown of TRIM24 or TRIM28 affected the reciprocal protein levels, as well the levels of TRIM33. In no case the individual protein knock-down altered the amounts of the other TIF1 members. Similarly, knock-down of TRIM33 had no effect on the levels of either TRIM24 or TRIM28, while it markedly increased IN amount, thus confirming the previous findings (Fig. 3d). We conclude that TRIM33 is the only TIF1 protein regulating HIV-1 IN levels.

Next we assessed whether TRIM33 silencing stabilized HIV-1 IN after blocking protein synthesis with cycloheximide. In these experiments, we transiently knocked-down TRIM33, Pin1, or LEDGF/p75 24 h before cell transfection with Flag-IN. After additional 48 h, we treated cells with cycloheximide for 0, 30, 60, and 90 min IN stability was examined by immunoblotting with anti-Flag antibody. In TRIM33-depleted cells, the basal levels of IN were increased and its rate of disappearance markedly slowed down, indicative of protein stabilization (~75% of protein still present after 90 min of CHX treatment vs. <20% in control cells; Fig. 3e for representative blots and Fig. 3f for quantification). Depletion of LEDGF/p75 showed an opposite effect, albeit more modest, consistent with the protective function of this factors on IN degradation. The results of these experiments indicate that TRIM33 is a major determinant of IN stability.

Next we wondered whether TRIM33 was capable to also modulate the stability of MLV IN or exclusively acted on the HIV-1 protein; in this set of experiments we also analyzed the effect of other HIV-IN-stabilizing cellular factors (Pin1, LEDGF/p75, and Ku70) on MLV IN. Cells were transfected with siRNAs against the individual factors under investigation together with plasmids expressing either Flag-tagged HIV-1 or MLV IN proteins. Forty-eight hours post transfection, total IN levels were examined by immunoblotting with anti-Flag antibody. HIV-1 IN levels increased upon TRIM33 silencing and decreased upon silencing of the stabilizing factors (Fig. 3g top panel for a representative immunoblot and Fig. 3h for quantification). In the case of MLV IN, neither knock-down of TRIM33 increased nor silencing of LEDGF/p75 or Ku70 decreased protein level, while Pin1 knock-down even augmented it (Fig. 3g bottom panel and Fig. 3h).

The HIV-1 IN protein, which normally carries an N-terminal phenylalanine, is a target of the proteasome-dependent N-rule protein degradation pathway^3^. To assess whether TRIM33 might be involved in this process, we expressed untagged IN by transfecting HeLa cells with bicistronic plasmids co-expressing EGFP and various IN variants carrying different N-terminal amino acids^3^ (Supplementary Fig. 3a). As expected, the levels of variants carrying phenylanine or histidine as the first amino acid were markedly reduced by proteasomal degradation, as evidenced by the capacity of MG132 to rescue this phenotype (Supplementary Fig. 3b). Depletion of TRIM33 increased the levels of all variants, but did not revert the effect of the N-terminal rule pathway on the phenylanine and histidine-carrying IN proteins. These results indicate that TRIM33 affects IN stability with a mechanism that is additional to that of the N-terminal rule, also consistent with binding of TRIM to a portion of IN different from the N-terminal domain (cf. later).

### TRIM33 E3 ligase marks IN for degradation via poly-ubiquitination

**Fig. 4:**
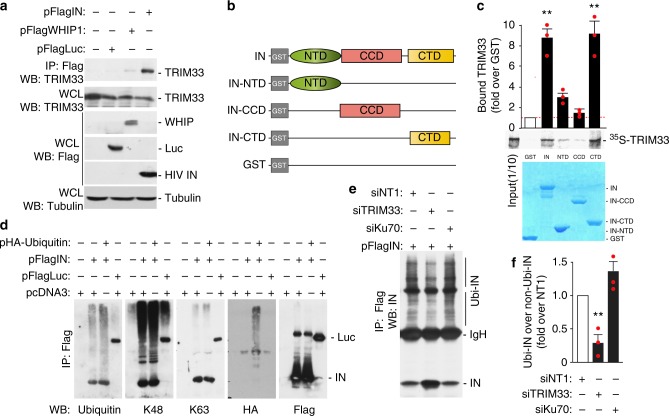
Cellular TRIM33 binds HIV-1 integrase and regulates its degradation by poly-ubiquitination. **a** Endogenous TRIM33 interacts with HIV-1 IN in vivo. HEK293T cells were transfected with the plasmids indicated on the top of the panel and immunoprecipitated with anti-Flag M2 beads, followed by immunoblotting with anti-TRIM33 antibody (upper blot). The lower three blots show the levels of expression of the transfected proteins in cell lysates. **b** Schematic representation of the HIV-1 IN domains used for the pull-down assay in panel **c**. NTD N-terminal domain, CCD catalytic core domain, CTD C-terminal domain. **c** TRIM33 binds the HIV-1 IN C-terminal domain (CTD) in in vitro pull-down assay. The indicated fragments of the HIV-1 IN protein fused to GST or GST alone were incubated with in vitro translated [^35^S]-TRIM33, extensively washed, and then resolved by SDS-PAGE. The middle pictures shows the gel exposed to a phosphoimager from a representative experiment along with the quantification of the amount of bound proteins expressed as a percentage of radiolabeled input. The lower panel shows the protein gel stained with Coomassie blue to visualize proteins. Quantification in the upper graph reports mean ± SEM; *n* = 3 independent experiments; **P < 0.01; one-way ANOVA. **d** HIV-1 IN is degraded through Lys48-linked polyubiquitination. HEK293T cells were transfected with Flag-IN and HA-Ubiquitin for 48 h, as indicated. The ubiquitination profile of IN was visualized after immunoprecipitation with anti-Flag M2 beads using anti-ubiquitin, anti-K48, anti-K63, anti-HA, and anti-Flag antibodies. **e** TRIM33 Inhibition reduces IN polyubiquitination. HEK293T cells were co-transfected with Flag-IN and HA-Ubiquitin for 36 h, as indicated. IN polyubiquitination was visualized after immunoprecipitation with anti-Flag M2 beads using an anti-IN antibody. **f** Quantifications of the amount of polyubiquitinated IN protein (Ubi-IN) in whole cell extracts, normalized over total IN. Experiments were performed as in panel **e**

TRIM33 belongs to the RING type ubiquitin E3 ligase family, the members of which ubiquitinate substrate proteins by directly binding^17,47^. The protein has a predominately nuclear localization, both soluble and chromatin-bound, however a significant portion of it was is detected in the cytoplasm (Supplementary Fig. 4). Recent information indicates that cytoplasmic TRIM33 is also present in CD4+ T lymphocytes^48^; here, the protein can interact with incoming HIV-1 IN. Thus, we wanted to further explore the interaction between TRIM33 and IN by co-immunoprecipitation studies. A Flag-IN expressing plasmid, or control plasmids expressing Flag-Luciferase or Werner helicase interacting protein1 (WHIP1) were transfected into HEK293T cells. WHIP1 contains a zinc-finger domain and is found in both nucleus and cytoplasm. Similar to TRIM33, WHIP1 also binds chromatin^49,50^. The Flag-tagged proteins were immunoprecipitated with anti-Flag M2 magnetic beads and the amount of endogenous TRIM33 in the precipitated immunocomplexes was examined by western blotting with an anti-TRIM33 antibody (Fig. 4a). We found that endogenous TRIM33 bound IN; both transfected proteins and endogenous TRIM33 were well expressed inside the cells.

To map the domain of IN that mediates binding to TRIM33, we performed pull-down assays using GST-tagged proteins corresponding to full-length IN or segments of the protein corresponding to its N-terminal domain (NTD), catalytic core domain (CCD) and C-terminal domain (CTD); GST alone served as a negative control (Fig. 4b). Binding of these proteins to in vitro translated, [^35^S]-labeled TRIM33 revealed binding to full length IN and to the IN CTD domain (>8.5% binding; *P* < 0.01 in both cases), but not to the other domains of the protein (<3% binding; Fig. 4c). All the GST fusion proteins were expressed and purified at comparable levels for the GST-pull down binding assays (Coomassie blue-stained gel at the bottom of Fig. 4c).

Next we examined IN ubiquitination in HEK293T cells transfected with Flag-tagged IN together with a plasmid expressing HA-tagged ubiquitin, followed by analysis of the IN ubiquitination profile in samples immunoprecipitated with Flag M2 beads. We found that ubiquitin molecules are mainly linked to IN through lysine 48 (K48), rather than K63, consistent with the notion that K48-polyubiquitinated IN is further recognized by the cellular ubiquitin/proteasome system for protein degradation (Fig. 4d). These results were further validated by co-expressing various HA-tagged ubiquitin mutants (K48, K48R, and KO/no lysine) together with Flag-tagged IN in HEK293T cells, followed by IN immunoprecipitation and visualization of its modification using HA and anti-K48 antibodies. Here again we found increased levels of ubiquitinated IN in cells overexpressing K48-ubiquitin compared to those transfected with a K48R or KO-no lysine ubiquitin mutants (Supplementary Fig. 5a). These results are all consistent with the conclusion that K48-linked polyubiquitination mainly leads to HIV-1 IN degradation.

Next, we examined polyubiquitination of IN in TRIM33-depleted cells, as well as in Ku70-depleted cells, in the absence of proteasome inhibitor. We found that the knockdown of TRIM33 reduced IN polyubiquitination (Fig. 4e, f). Of note, in these experiments the knockdown of TRIM33 also determined a significant accumulation of non-polyubiquitinated protein, in line with its stabilizing effect on the protein.

We also assessed the involvement of different E2 conjugating enzymes in IN poly-ubiquitination. These experiments were performed using purified recombinant Flag-tagged TRIM33 (obtained in mammalian cells; Supplementary Fig. 5b) and recombinant GST-IN (obtained in E. coli, Supplementary Fig. 5c), using an E2 scan kit analyzing the effect of 34 different E2 ligases. We found that multiple E2s are capable of TRIM33-dependent IN ubiquitination, including 2E1, 2E2, 2E3, 2F, 2G1, 2H, and 2Q (Supplementary Fig. 5d).

### Mapping the TRIM determinants involved in IN regulation

To identify which TRIM33 domain is important for its interaction with IN, we co-expressed EGFP-tagged IN together with various Flag-tagged TRIM33 variants carrying deletions in the PHD/Bromodomain, RBCC/TRIM domain or both (ΔPHD/Bromo, ΔTRIM and Linker respectively; Supplementary Fig. 6a). After recovery of the TRIM33 proteins using anti-Flag M2 magnetic beads, we tested the immunoprecipitates for the presence of IN. We found that IN bound to wild-type TRIM33 and the ΔPHD/Bromo deletant, but not to the other mutants (Supplementary Fig. 6b). All the proteins were equally expressed in the cells. We conclude that integrity of the RBCC/TRIM domain at the N-terminus of TRIM33 is essential for its interaction with IN.

**Fig. 5:**
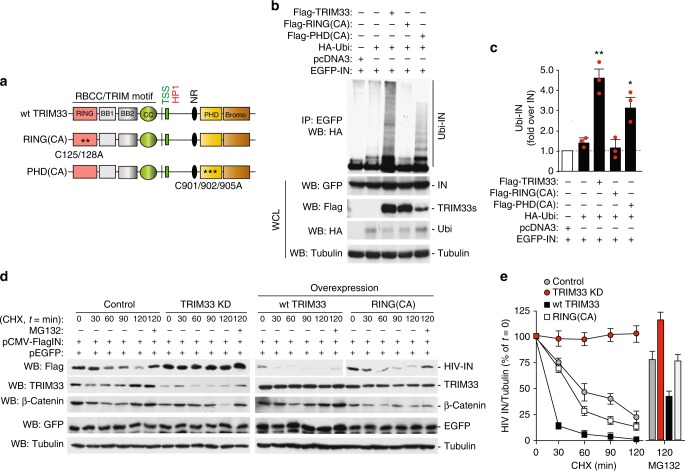
Mapping the TRIM determinants determining integrase stability. **a** Schematic representation of the TRIM33 protein and its catalytically inactive mutants in the RING and PHD domains [RING(CA) and PHD(CA) respectively]. **b** Integrity of the TRIM33 RING motif is essential for HIV-1 IN ubiquitination. HEK293T cells were transfected with combinations of plasmids expressing HA-ubiquitin, EGFP-IN, Flag-TRIM33 and its catalytically inactive mutants (RING and PHD), and pcDNA3 as an empty vector. Cells were harvested after 5 h treatment with proteasome inhibitor and cell lysates were immunoprecipitated with anti-GFP antibody. Ubiquitin-conjugated IN (Ubi-IN) was detected by immunoblotting with anti-HA antibody (upper picture). The lower pictures show the levels of expression of the transfected plasmids in whole cell lysates control expression of transfected proteins after immunoblotting with the respective antibodies. **c** Quantifications of the amount of ubiquitinated IN protein (Ubi-IN) in whole cell extracts, normalized over total IN. Experiments were performed as in panel **b**. Data are mean ± SEM; *n* = 3 independent experiments; *P < 0.05; **P < 0.01; one-way ANOVA. **d** Representative immunoblot showing the stability of HIV-IN in HeLa cells stably expressing an shRNA against TRIM33 (TRIM33 KD) and transfected to overexpress wild-type TRIM33 or the C125A/C128A catalytically inactive (RING(CA). Cells were co-transfected with Flag-IN and EGFP, as transfection and specificity control; 48 h later, the levels of IN were assessed by anti-Flag immunoblotting. Tubulin served as a loading control; β-catenin was used to confirm effect of MG132 treatment; knock-down and overexpression of TRIM33 variants were verified by anti-TRIM33 immunoblotting. **e** Quantification of the effects of TRIM33 knock down and overexpression on the levels of HIV-1 IN. Results of three independent experiments (mean ± SEM) performed as in panel **d** are expressed, after normalization for Tubulin, as a percentage of time = 0 for each treatment

TRIM33 harbors two catalytically active domains, RING and PHD, which can post-translationally modify the target proteins by conjugating them with ubiquitin and ubiquitin-like molecules^16,17,51^. To understand which of these two domains is responsible for IN ubiquitin-mediated degradation, we co-expressed EGFP-IN and HA-Ubiquitin with Flag-tagged wild-type TRIM33 or two catalytically inactive mutants bearing amino acid substitution in either of these domains, namely RING(C125A/C128A) and PHD(C901A/C902A/C905A); Fig. 5a. Forty-eight hours later, cells were treated with MG132 for 5 h. Then IN was immunoprecipitated from cell lysates with an anti-GFP antibody and the ubiquitinated forms of the proteins were visualized by immunoblotting with an anti-HA antibody (Fig. 5b for a representative immunoblot and Fig. 5c for quantification). Wild-type TRIM33 increased IN poly-ubiquitination, an effect that was abolished by the RING domain point mutations; mutations in the PHD domain, instead, had no apparent effect. Both TRIM33 mutants, however, were equally effective as the wild-type protein in binding to IN in GST pull-down assays (Supplementary Fig. 6c).

We also examined HIV-1 IN protein stability in HeLa cells stably transfected to express an shRNA against TRIM33 (TRIM33 KD), the wild-type TRIM33 protein or the catalytically inactive RING(CA) mutant. Analysis of protein levels was performed at different times after treatment with cycloheximide. We found that stable depletion of TRIM33 stabilized IN, consistent with our previous findings, while overexpression of the wild-type protein cDNA, but not that of its RING mutant, accelerated its degradation (representative blot and quantification in Fig. 5d, e respectively). Treatment with the MG132 proteasome inhibitor rescued the effect of wild-type TRIM33 overexpression on IN levels. No effect of any of the treatments was observed on simultaneously transfected EGFP protein, remarking the specificity of the findings for IN.

Taken together, these results corroborate the conclusion that TRIM33 is a novel HIV-1 IN E3 ligase that binds the IN carboxy-terminal domain and determines its poly-ubiquitination, pending integrity of its RING domain.

### TRIM33 inhibits HIV-1 infection at the viral DNA integration step

We wanted to understand the role of TRIM33 in HIV-1 infection. First, we assessed efficiency of single-round infection of HeLa cells that had been depleted of TRIM33, with a VSV-G-pseudotyped HIV-1 vector carrying the luciferase gene under the control of the HIV-1 LTR (clone NL4-3.Luc.R-E-; Supplementary Fig. 7a). Forty-eight hours after infection of cells in which TRIM33 had been silenced, luciferase activity (Supplementary Fig. 7b) and, more markedly, the levels of integrated HIV-1 DNA (Supplementary Fig 7c) were elevated, while those of 2LTR circles markedly reduced (Supplementary Fig. 7d). In contrast, downregulation of either Pin1 or LEDGF/p75 decreased both the amount of integrated provirus and luciferase activity, while 2LTR formation increased only in LEDGF/p75-depleted cells. In TRIM33-knock down cells, both early and late HIV reverse transcripts were also slightly increased (Supplementary Fig. 7e, f respectively), likely because of the effect of IN on the efficiency of reverse transcription^52^. We verified that the downregulation of the investigated proteins by siRNAs in these experiments was effective (>90% decrease; representative experiment in Supplementary Fig. 7g).

**Fig. 6:**
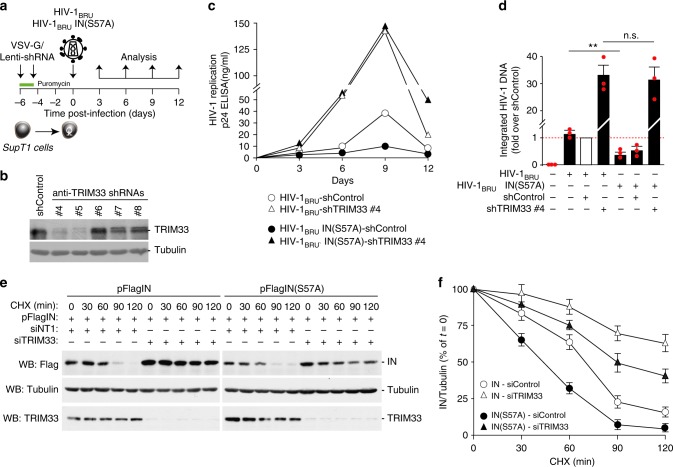
TRIM33 inhibits HIV-1 infection. **a** Analysis of the effect of stable knockdown of TRIM33 in multiple round infection with wild-type HIV-1. SupT1 cells were first transduced with lentiviral vector expressing different anti-TRIM33 shRNAs; after selection with puromycin, cells were infected with wild-type HIV-1_BRU_ or HIV-1_BRU_IN(S57A), followed by analysis of infection over time by p24 ELISA. **b** Western blotting analysis showing endogenous TRIM33 levels in SupT1 cells transduced with lentiviral vectors expressing the indicated shRNAs and selected with puromycin. Tubulin was used as a loading control. **c**. Stable knockdown of TRIM33 enhances wild type HIV-1 replication and rescues replication of the IN(S57A) mutant. The graph show the levels of p24 in control Sup1 cells or cells previously transduced with anti-TRIM33 shRNA #4, which efficiently knocks down TRIM33 expression, after infection with the two viruses. The results shown are representative or four independent experiments. **d** Quantification of the levels of integrated HIV-1 DNA in SupT1 cells, expressing an shRNA control or shRNA #4 against TRIM33, and infected with wild-type HIV-1_BRU_ or HIV-1_BRU_IN(S57A), at day 3 after infection. Data are mean ± SEM; *n* = 3 independent experiments; **P < 0.01; n.s. not significant; one-way ANOVA. **e** Silencing of TRIM33 protects both wild-type IN and mutant IN(S57A) from degradation. Expression plasmids for the two IN proteins were transfected into HeLa cells in which TRIM33 was knocked down by RNAi; cells were then treated with cycloheximide 30 µg/ml for the indicated time points prior to lysis. The panel shows a representative immunoblots using antibodies against IN, TRIM33 and tubulin as a loading control. **f** Quantification of IN levels in the presence of cycloheximide for the indicated time points in the whole cell extracts of SupT1 cells treated with siRNAs against TRIM33 or not targeting and transfected with either wt IN or IN(S57A). After normalization for Tubulin, data (mean ± SEM; *n* = 3 independent experiments) are expressed as a percentage of time = 0 for each treatment

Supported by these findings, we wanted to assess the effect of permanent TRIM33 knockdown on wild-type HIV-1 infection in T cells (experimental scheme in Fig. 6a). For this purpose, we generated five VSV-G-pseudotyped lentiviral vectors, each one expressing a different shRNA targeting TRIM33 (shRNAs #4, #5, #6, #7 and #8) and carrying a puromycin-resistance gene to selected transduced SupT1 cell clones. Vectors carrying shRNAs #4 and #5 were highly effective in downregulating the protein (representative blot in Fig. 6b; >80% of protein reduction). A lentiviral vector carrying a control shRNA, submitted to the same procedure, served as a control in these experiments.

Next, we transduced SupT1 T cells expressing shRNA #4 or shRNA #5 against TRIM33 and, after 6 day-puromycin selection, we infected these cells with wt HIV-1_BRU_; infection was monitored over a course of 12 days by measuring the amounts p24 viral protein in the cell culture supernatant. Infection was markedly increased in cells in which TRIM33 was knocked down (shown for anti-TRIM shRNA #4 in Fig. 6c). The negative effect of TRIM33 was also confirmed in single-round HIV-1_NL4-3.Luc.R-E-_ infection of the shRNA-transduced cells, in which luciferase activity was selectively increased in clones #4 and #5 in which the proteins had been knocked down (experimental scheme and results of luciferase acitivity in Supplementary Fig. 7h, i respectively). On the contrary, overexpression of the TRIM33 cDNA significantly decreased infection with this molecular clone (Supplementary Fig. 7i, j for luciferase activity and levels of TRIM33 in the transfected cells, respectively).

Our previous work had shown that an HIV-1 virus carrying the IN(S57A) point mutation, which severely hampers IN stability, is markedly less infectious^15^. Thus, we wondered whether the knock-down of TRIM33 might revert infectivity of this molecular clone. By analyzing T-cell infection with HIV-1_BRU_IN(S57A), we indeed confirmed that this clone is markedly less infectious than wt HIV-1_BRU_ in control cells, however we observed that its infectivity was completely rescued by the TRIM33 knock-down (shown for SupT1 cells carrying anti-TRIM33 shRNA #4 in Fig. 6c; again, superimposable results were obtained for shRNA #5). Impairment of HIV-1_BRU_IN(S57A) infection and rescue by TRIM33 downregulation was also observed by analyzing the levels of integrated DNA by Alu-LTR at day 3 after infection (Fig. 6d). In these experiments, TRIM33 known down resulted in similar levels of HIV-1 integrated DNA in cells infected with wt virus or the mutated IN clone.

We found that mutant IN(S57A) bound to TRIM33 equally well as the wild-type protein in immunoprecipitation assays (Supplementary Fig. 7l; however poly-ubiquitination of this protein was significantly enhanced (representative ubiquitin blot in Supplementary Fig. 7m and quantification in Supplementary Fig. 7n), consistent with increased protein degradation. Transient depletion of TRIM33 by RNAi resulted in markedly increased stability of the IN(S57A) mutant (estimated half-life: <45 min and >90 min in the absence and presence of TRIM33 knockdown, respectively; Fig. 6e, f for representative images and quantification, respectively).

Collectively, these results indicate that depletion of TRIM33 markedly augments HIV-1 infection by increasing integration of the viral genome into the host cell DNA and is sufficient to revert the phenotype of HIV-1_BRU_IN (S57A) by increasing stability of the mutant IN protein.

**Fig. 7:**
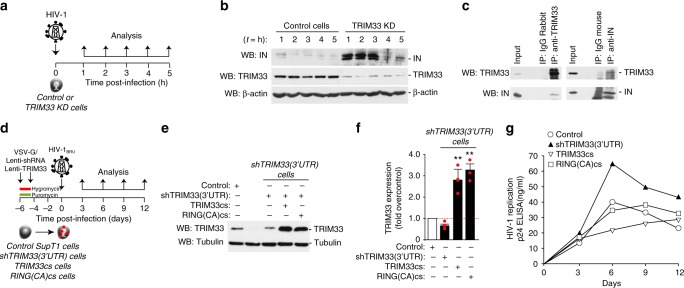
Interaction of TRIM33 with integrase from infectious HIV-1. **a** Analysis of IN protein levels in TRIM33-knockdown (TRIM33 KD) cells during HIV-1 infection. Cells stably expressing an anti-TRIM33 shRNA were infected for 1 h with HIV-1; at the indicated time points after infection, cells were washed with PBS and the levels of IN were analyzed. **b** Western blot showing IN levels in control and TRIM33 depleted cells Beta-actin was used as a loading control; TRIM33 downregulation was verified using an anti-TRIM33 antibody. **c** TRIM33 interacts with IN during HIV-1 infection. SupT1 cells were infected with HIV-1 in the presence of MG132. Either TRIM33 or IN were immunoprecipitated with the respective antibodies, followed by immunoblotting to test the presence of the reciprocal protein. **d** Analysis of the effect of stable knockdown and overexpression of TRIM33 variants in multiple round infection with wild-type HIV-1. SupT1 cells stably expressing an hRNA targeting the TRIM33 3′-UTR (selected after transduction with a lentiviral vector and selection for puromycin) were transduced with lentiviral vectors expressing the TRIM33 coding sequence (cs) or the coding sequence of the RING(CA) mutant. After selection with hygromycin, cells were infected with wild type HIV-1_BRU,_ followed by analysis of infection over time by p24 ELISA. **e** Western blotting analysis showing TRIM33 levels after transduction with shRNA targeting 3′-UTR of TRIM33 alone or after transduction of wild-type or mutant TRIM33 (TRIMM33cs or RING(CA)cs. Tubulin was used as a loading control. **f** real-Time RT-PCR analysis showing TRIM33 expression after transduction with the shRNA targeting the 3′-UTR of TRIM33 alone or after transduction of wild type or mutant TRIM33. **g** Stable knockdown of TRIM33 enhances wild-type HIV-1 replication, while overexpression of its catalytically active cDNA reduces it. The graph shows the levels of p24 in control Sup1 cells or cells previously transduced with anti-TRIM33 shRNA targeting the 3′-UTR of the protein, with or without transduction with a lentiviral vector expressing the coding sequence of wild type TRIM33 (TRIM33cs) or that of the catalytically inactive RING(CA) mutant (RING(CA)cs), after infection with wild-type virus. The results are representative of three independent experiments

We wanted to assess the role of TRIM33 in regulating degradation of IN carried by HIV-1 during infection. We thus infected TRIM33 KD cells, which stably express an anti-TRIM33 shRNA, with HIV-1 (25 μg of p24 per million of cells), followed by analysis of IN levels by immunoblotting for the subsequent 5 h (experimental scheme in Fig. 7a). Incoming HIV-1 IN degradation in the early hours after infection was markedly reduced in cells expressing the TRIM33 shRNA (Fig. 7b). In similar experimental conditions, we also investigated binding between endogenous TRIM33 and virion-associated IN in control infected cells. After 4 hour HIV-1 infection in the presence of proteasome inhibitor, interaction between IN and TRIM was assessed by immunoprecipitation in whole cell lysates. We found that both TRIM33 and IN co-immunoprecipitated with the respective partners during viral infection (Fig. 7c).

Finally, we also wanted to examine viral replication in cells in which endogenous TRIM33 was stably knocked down and then restored through expression of its either its wild type or mutant cDNA. For this purpose, we generated a lentiviral vector expressing an shRNA targeting the 3′UTR of endogenous TRIM33 (shTRIM33(3’UTR)) and used this vector to generate a stable SupT1 T cell line by selection for puromycin. This line was then transduced with one of either two additional lentiviral vectors, carrying the resistance gene for hygromycin, expressing the coding sequences for either wild type TRIM33 or its catalytically inactive RING mutant (TRIM33cs and RING(CA)cs respectively); both these cDNAs were devoid of the 3′ UTR and thus insensitive to the shRNA (scheme in Fig. 7d). After 7 days double antibiotic selection, we verified proper expression of the TRIM proteins in the different clones (representative western blot and RT-PCR quantification in Fig. 7e, f and respectively) and then infected these cells with wt HIV-1_BRU_; infection was monitored over a course of 12 days by measuring the amounts p24 viral protein production. Infection was significantly increased in cells which stably expressed the anti-TRIM33 shRNA. Overexpression of the TRIM33cs decreased HIV-1 infection while this effect was blunted by expression of its catalytically inactive mutant (Fig. 7g).

Taken together, these results are consistent in showing that cellular TRIM33 binds IN from infectious HIV-1 and regulates viral replication by promoting its degradation.

## Discussion

High throughput screening of a human siRNA library targeting all known components of the ubiquitin conjugation system has revealed that the TRIM33 RING finger E3 ligase is responsible for HIV-1 IN degradation and thus acts an inhibitory factor for HIV-1 infection by preventing viral DNA integration. Thus, TRIM33 adds to the group of cellular proteins that counteract different steps of the virus life cycle, including capsid disassembly (rhesus TRIM5 proteins), prevention of reverse transcription (SAMHD-1), viral cDNA nuclear import (MxB), release of nascent viral particles (Tetherin/BST-2), viral RNA deamination (members of the APOBEC3 (A3) family; reviewed in ref. ^53,54^) and virion infectivity (SERINC3 and SERINC5^55,56^). Consistent with an inhibitory role at the pre-integration step, overexpression of TRIM33 decreased the levels of integrated viral DNA while its knock-down had an opposite effect. In addition, absence of TRIM33 rescued infectivity of an HIV-1 molecular clone carrying a substitution of serine 57 to alanine, a mutation that destabilizes integrase activity while leaving earlier steps in the virus life cycle unaffected, including uncoating and reverse transcription^15^.

In our screening for siRNAs that rescued HIV-1 IN degradation, TRIM33 was the only factor (i) that scored in the top 10 positions in the duplicate screenings, (ii) that was the most effective in increasing IN levels when knocked down using an siRNA pool and (iii) for which three individual siRNAs out of four reproduced the phenotype of the siRNA pool after deconvolution. Three other siRNA pools (against FBOX28, DTX2, and UBE2J2) also increased IN levels significantly, but deconvolution revealed that either the individual siRNAs composing the pools were not active against their respective targets or that their effect did not increase IN levels. UBE2J2 also scored negative in the E2 scan kit in which we analyzed the effect of all E2 enzymes.

In the cases of FBXO42, NHLRC1/Malin, and TRIM72 we could not assess the levels of individual protein silencing since none of the available antibodies were effective; however, the siRNA pools against these proteins did not increase IN levels by western blotting. No effect on IN was also the case for the RNF31, RNF125/TRAC1, and RFPL3 siRNAs. As far as the last of these three factors is concerned, published evidence indicates that RFPL3 stimulates HIV-1 PIC integration activity in an in vitro integration assay^29^, thus acting in an opposite manner as predicted for a destabilizing, IN-specific E3 ligase.

Past work using a yeast two hybrid system has shown that IN can bind the von Hippel–Lindau binding protein 1 (VBP1), a subunit of the prefolding chaperone, which bridges interaction between IN and the cullin2 (CUL2)-based von Hippel–Lindau (VHL) ubiquitin ligase^57^. In our assay conditions for IN stability, however, siRNAs against all components of the complex (VBP1, VHL, and CUL2) were ineffective in increasing IN levels, while VHL scored neutral in our original screening with all E3 ligases. Downregulation of VBP1 or VHL, however, has been reported to markedly decrease, and not increase, HIV-1 replication, since this interaction would be expected to occur at a step when viral cDNA integration has already occurred and to be necessary for efficient transcription of viral genes^57^. Thus VHL, in any case, would not qualify as the E3 ligase primarily responsible for IN stability and thus efficiency of viral cDNA integration, as instead is the case for TRIM33.

TRIM33 (TIF1γ) belongs to the TRIM (tripartite motif) family of proteins, which display potent antiviral activity against several viruses^39^. The protein was originally discovered as its cDNA, under low stringency conditions, cross-hybridized with a probe corresponding to a portion TRIM24 (TIF1α), the founding member of the TIF1 family^42^. In mice and humans, TRIM33 acts as a tumor suppressor in different tissues, including hematopoietic compartment^58^, liver^59^ and pancreas^60^, and as a major regulator of TGFβ signaling in development. In particular, TRIM33 interacts with TGFβ receptor-activated, phosphorylated SMAD2/3 and competes with formation of a heterodimer with SMAD4, an essential step in TGFβ signaling^61^. Additionally, TRIM33 also mediates ubiquitination of SMAD4 attenuating the transcriptional response to TGFβ signaling, thus favoring ectoderm specification^62,63^. TRIM33 was also shown to be recruited at sites of DNA damage in a PARP1- and ALC1- dependent manner^64^ and to regulate cell cycle progression^65,66^. At the chromatin level, TRIM33 behaves as a transcriptional co-suppressor^49,67^.

Our work indicates that TRIM33 suppresses HIV-1 infection by promoting ubiquitin/proteasome-mediated degradation of IN, thus preventing proviral formation. TRIM33 has mainly nuclear localization, but a portion of it can also be found in the cytosol. Thus, we speculate that it might encounter the incoming IN in both compartments, prior to proviral formation. HIV-1 is not a unique target for TRIM33 antiviral function, since the protein also limits adenoviral infection by blocking early and late gene expression, an activity that is counteracted by the viral E4-ORF3 protein^68,69^.

Studies using recombinant proteins have shown that the coiled-coil region of TRIM33 mediates heterodimerization of the protein with TRIM24^70^; in addition, in mouse hepatocytes, these two proteins are also found in complex with the third member of the TIF1 family, TRIM28^59^. Of interest, our own past work has revealed that TRIM28/KAP1 selectively binds IN post-translationally modified by acetylation and that, in complex with HDAC1, curtails viral infection by promoting IN deacetylation^71^. Given these observations and considering the high similarity in domain organization and sequence among the three TIF1 members, we wondered whether TRIM24 or TRIM28 might also affect IN stability. We found, however, that TRIM33 was the only one of the three proteins to be effective. In addition, no effect of TRIM33 knock-down on the levels of either of the two other members was detected.

TRIM33 belongs to RING-type E3 ligases, which bind both their target proteins and ubiquitin-loaded E2-conjugating enzyme and catalyze transfer of ubiquitin to the target^17,47^. Indeed, we found that IN and TRIM33 interacted directly in vitro by pull-down assays and in vivo by co-immunoprecipitation; integrity of the C-terminal domain of IN and the RING domain of TRIM33 were essential for this interaction to occur. Consistent with this information, deletion and point mutations in the TRIM33 RING domain both impeded binding and blocked IN ubiquitination respectively; mutations in the PHD domain were ineffective. Thus, HIV-1 IN adds to the list of cellular proteins the stability of which is regulated by the E3 ubiquitin ligase activity of TRIM33, capable to induce both ubiquitination (for SMAD4^62^ and LDB1^72^) and SUMOylation (for SnoN1^73^).

In addition to their substrates, RING-type E3 ligases also directly bind their cognate E2 conjugating enzymes. Approximately 35 of these E2 enzymes are predicted to exist, all of which contain a catalytic (Ubc) domain with an active site cysteine residue^74^. Known members of the TRIM family specifically interact with the D and E subclasses of E2 conjugating enzymes, except TRIM9, which interacts with UBE2G2 and TRIM32 with UBE2V1/V2^75^. By scanning the effect of 34 purified E2 enzymes in the presence of TRIM33 we found that multiple E2s were able to assist this E3 ligase in the poly-ubiquitination of IN. This redundancy well explains why no specific E2 enzymes were detected in our primary siRNA screening for IN stability.

The effect of TRIM33 in determining IN poly-ubiquitination and degradation was specific for the HIV-1 enzyme. In contrast, the Moloney MLV IN was long-lived, relatively insensitive to even high doses of proteasome inhibitor and unaffected by TRIM33 downregulation. In addition, sensitivity of HIV-1 IN to TRIM33-mediated proteasomal degradation was increased by silencing of LEDGF/p75, Ku70, and Pin1, while no protective effect of these factors was detected for MLV IN. LEDGF/p75 is a selective partner of HIV-IN^5,12,13^, while Ku70 was shown to bind both HIV-1 IN^14^ and MLV IN^76^. For both factors, binding to HIV-1 IN was also shown to prevent its proteasomal degradation^7,14^. Thus, we can speculate that the protective effect of these factors on HIV-1 IN stability is exerted by masking binding to TRIM33, a conclusion that can be tested experimentally using recombinant factors.

In the case of Pin1, this enzyme isomerizes phosphorylated HIV-1 IN and this conformational change increases IN stability^15^. Again, it can be postulated that cis-trans isomerization of HIV-1 IN alters TRIM33 recognition. Finally, of additional potential interest for future research, our experiments also reveal that knock-down of Pin1 increased MLV IN, opposite of HIV-1 IN, possibly hinting to a still undefined role of this isomerase on MLV stability.

The molecular effects exerted by TRIM33 on the stability of HIV-1 IN have significant impact on the infectivity of HIV-1 virions. Overexpression of the protein curtails HIV-1 infection, while its silencing enhances it, as proven by both single-round infections or standard infections with wild type virus. Of note, our results show that TRIM33 knock-down reverts the phenotype of an HIV-1 virus carrying the IN(S57A) point mutation, which our past work had shown to be less infectious because its IN is more prone to proteasomal degradation^15^. This finding represents a further indication of the relevance of the TRIM33 E3 ligase in the HIV-1 life cycle.

Finally, we have previously reported that HIV-1 integrates its DNA in euchromatic regions in correspondence to the nuclear periphery and have suggested that this peculiar localization might be the inevitable consequence of the relatively short life of viral IN, which would prevent effective inward diffusion of the virus^77^. Based on the results shown here, we predict that TRIM33 knock down would allow the virus to also reach inner compartments in the nuclear interior, a hypothesis that will be tested in future experiments.

## Methods

### Cells and antibodies

HeLa (ATCC, CCL-2) and HEK293T (TaKaRa Bio USA no. 632273) cell were maintained in DMEM supplemented with 10% FBS and gentamycin (Sigma). Jurkat and SupT1 cells (NIH Research and Reference Reagent Program) were maintained in RPMI with 10% FBS. Primary human CD4+ T cells were isolated by Ficoll-Percoll gradient separation^78^ from blood followed by purification with CD4 MicroBeads (Miltenyi Biotec). CD4+ T Cells were activated with a cocktail of beads containing 4.5 × 10^5^ beads coated with CD3 and CD28 antibodies (Dynabeads Human T-Activator CD3/CD28 Dynal/Invitrogen), and plated in complete medium with IL-2 (30 U/ml, Sigma-Aldrich) for 4 days at 37 °C.

The following antibodies were used: anti-IN (SantaCruz, sc-69721, dilution 1:1000), anti-GFP (SantaCruz, sc-8334, 1:500), anti-β-catenin (SantaCruz, sc-70509, 1:1000), anti-JNK1/2 (SantaCruz, sc-571, 1:500), anti-UBE2J2 (SantaCruz, sc-134139, 1:500), anti-DTX2 (SantaCruz, sc-101938, 1:500), anti-Pin1 (SantaCruz, sc-53924, 1:500), anti-Ku70 (Bethyl laboratories, 1:1000), anti-TRIM33 (Bethyl laboratories, A301-060A, 1:1000), anti-TRIM24 (Bethyl laboratories, A300-816A, 1:1000), anti-TRIM28 (Bethyl laboratories, A300-274A, 1:1000), anti-FBXO28 (Bethyl laboratories, A302-377A, 1:1000), anti-LEDGF/p75(Bethyl laboratories, A300-848A, 1:1000), anti-CUL2(Bethyl laboratories, A302-476A, 1:1000), anti-VHL(Cell signaling#2738, 1:1000), anti-RNF31(Abcam, ab85294, 1:1000), anti-RNF125(Abcam, ab74373, 1:1000), anti-TRIM33 (Cell Signaling, #13387, 1:1000), anti-Ubiquitin (Cell Signaling, #3933, 1:1000), anti-K48 (Cell Signaling, #8081, 1:1000) and anti-K63 (Cell Signaling, #5621, 1:100), anti-GFP (Molecular Probe Invitrogen, A6455, 1:1000), anti-RFPL3 (Life Span Biosciences, LS-B2822, 1:500), anti-PARP1(ENZO Life Sciences, ALX-210-302-R100, 1:1000) and anti-HA (Roche, 11867423001, 1:5000). Anti-Flag (F3165, 1:2000), anti-Flag-HRP (A8592, 1:5000), anti-Tubulin (T5168, 1:10,000) and anti-HSC70 (SAB3701436, 1:10,000) and anti-β-actin HRP (AC-15, 1:10,000) were all purchased from Sigma. Anti-phosphorylated IN antibodies were previously described^77^.

### Plasmids and vectors

EGFP-IN was provided by Anna Cereseto (University of Trento, Italy), pCS2-FlagTRIM33 and its variants were provided by Stefano Piccolo (University of Padova, Italy), Expression vectors for GST-IN full length and GST-IN truncated domains (N-terminus, Core and C-terminus) were previously described^15,79^. pFlag-IN CO was provided by Alan Engelman (Dana-Farber Cancer Institute, Boston, MA). pNL4-3.Luc.R-E- was a gift from Nathaniel Landau (New York University, NY, USA). The lentiviral vector expressing the TRIM33 cDNA was kindly provided by David A. Calderwood (Yale University School of Medicine, New Haven, USA); other TRIM33 lentiviral vectors were purchased from Open Biosystem (Huntsville, AL) and control pLKO.1based scramble lentiviral vector was purchased from Addgene. Molecular viral clones pBruINwt and PBruIN(S57A)mt were previously described^15^.

### siRNA screening

The siRNA library corresponding to all known human genes in the cellular ubiquitin-proteasome conjugation system (~598 target genes, siGENOME SMARTpools, 4 siRNAs per gene target) was purchased from Dharmacon, Thermo Scientific For the screening, siRNAs were transferred robotically from stock library plates to 96-well plates (PerkinElmer) leaving 2 columns empty for the addition of controls (buffer, non targeting siRNAs, siRNAs against UBC, Pin1, and JNKs). SiRNAs were transfected into HeLa cells using a standard reverse transfection protocol, at a final siRNA concentration of 50 nM. Briefly, the transfection reagent (Lipofectamine RNAiMAX, Life Technologies) was diluted in OPTI-MEM (Life Technologies) and added to the siRNAs arrayed on 96-well plates; 30 min later, 8.0 × 103 cells were seeded per well. Twenty-four hr after siRNA transfection, EGFP-tagged IN was transfected using a standard protocol, at a final concentration of 100 ng reporter plasmid per well. Briefly, the transfection reagent (FugeneHD, Promega) was diluted in OPTI-MEM (Life Technologies), mixed with the plasmid DNA, and added to the siRNA-transfected plates; after 48 h, cells were fixed and processed for immunofluorescence. Screening was performed in duplicate. The screening was performed at the ICGEB High-Throughput Screening Facility (http://www.icgeb.org/high-throughput-screening.html).

### Image acquisition and analysis

Image acquisition was performed using an ImageXpress Micro automated high-content screening fluorescence microscope (Molecular Devices) at a ×10 magnification; a total of 16 images were acquired per wavelength, well and replicate (~10,000–15,000 cells per well and replicate). Image analysis was performed using the ‘Multi-Wavelength Cell Scoring’ application module implemented in MetaXpress software (Molecular Devices). Briefly, nuclei were segmented based on Hoechst 33342 staining, and cells were then classified as positive or negative depending on the total area of GFP-IN staining.

### Immunoprecipitation and immunofluorescence

For immunoprecipitation, cell pellets were lysed in lysis buffer (50m Tris HCl pH 7.5,150 mM NaCl, 1 mMEDTA, 0.5% NP40) containing protease inhibitors (Roche). Protein concentration of the extracts was determined using the Bradford assay (BioRad). Anti-Flag M2 agarose beads (Sigma) and anti-GFP antibodies were incubated overnight at 4 °C with cell extracts (0.5–1 mg). Following incubation, Protein-A agarose beads (Santa Cruz) were used to bind the immunocomplexes, according to the manufacturer's instructions. After incubation, beads were washed 5 times with lysis buffer, and then processed for SDS-PAGE and immunoblotting using appropriate antibodies. For co-immunoprecipitation from HIV-1 infected cells, cells were lysed in cold lysis buffer (35 mM Tris HCl pH 7.5, 175 mM NaCl, 0.5 mM DTT, 1.25 mM MgCl_2_, and 0.05% NP-40) containing protease inhibitors. TRIM33 and IN antibodies were conjugated with Dynabeads™ Protein G (Invitrogen) and 3 mg of total protein lysates were used for immunoprecipitation according to the manufacturer’s instructions.

### In vivo ubiquitination assay

For in vivo ubiquitination, HEK293T cells were co-transfected with EGFP-IN, wild type TRIM33 and its mutants and HA-tagged ubiquitin plasmids by standard calcium phosphate method. After 30 h, cells were pretreated with the proteasome inhibitor MG132 before harvesting. Whole cell extracts were prepared in RIPA buffer - 50 mM Tris-HCl, pH 8.0, 150 mM NaCl, 1 mM EDTA, 1 mM PMSF, 0.5% NP40, protease inhibitor cocktail (Roche) - and 1.5 mg protein was immunoprecipitated with specific antibody. After immunoprecipitation, the samples were extensively washed and resolved by SDS-PAGE and analyzed by western blotting using appropriate antibodies.

### Cellular fractionation

HeLa cells were transfected with a Flag-IN expressing plasmid (2 µg/well) in 6-well plates. After 48 h, cells were treated with proteasome inhibitor for 4 h. Cells were then washed with 1XPBS, trypsinized, collected into tubes and washed with cold PBS. Cellular fractionation was performed using a subcellular protein fractionation kit as described by the manufacturer (Thermo Fisher). Fractionated samples were separated by SDS-PAGE and electro blotted to a nitrocellulose membrane. IN and TRIM33 subcellular distribution and different sub cellular markers (Tubulin and PARP1) were monitored by immunoblotting using antibodies against IN as anti-Flag, anti-TRIM33, -Tubulin, and -PARP1.

### Ubiquitin E2-conjugating enzyme screening

In vitro ubiquitin conjugation reactions were conducted in 20 μl containing UBE1, one of 34 different recombinant E2 enzymes, 2 mM ATP, 400 μM ubiquitin, purified recombinant TRIM33 E3 ligase and GST-IN at 37 °C for 1 h, according to the manufacturer’s instructions (Ubiquigent E2^scan^ kit, Cambridge, USA). CHIP E3 ligase/UBE2D4 were used as a positive control for the ubiquitination reaction, as it undergoes auto-ubiquitination. After 1 h, the reactions were stopped with SDS-PAGE sample buffer and resolved in 8% SDS-PAGE and analyzed by western blotting using anti-ubiquitin and anti-IN antibodies.

### Virus production and infection

Infectious viral stocks were produced by transfecting the plasmid expressing the viral genome into HEK293T cells by standard calcium phosphate method and collecting supernatants after 48 h. The molecular clone for HIV-1_BRU_ corresponds to that of HIV-1_LAI_. Viral production was quantified in the supernatants for HIV-1 p24 antigen content using the Innotest HIV antigen mAB kit (Innogenetics N.V. Gent, Belgium). Before infection, the viral stock was treated with 40 U/ml DNAse I (Life Sciences) for 1 h at 25 °C. HeLa/SupT1/primary CD4+ T cells (1x10^6^) were then infected with 1 μg of p24 for 4 h in the presence of polybrene (Sigma). After infection, cells were kept in culture at 1 × 10^6^ cells/ml in complete medium supplemented with IL-2. At days 3, 5, 7, and 10 post-infection, media and IL-2 were replaced and cells were kept at a density of 1 × 10^6^/ml. The Env- molecular clone pNL4-3.Luc.E^-^R^-^, a kind gift from Nathaniel Landau, harbors a frameshift mutation introduced near the 5′ end of envelope gene^80^ and performs a single-round infection once pseudotyped with the Vesicular Stomatitis Virus–G (VSV-G) protein.

Lentiviral particles were produced in HEK293T cells by transfecting 20 μg plasmid expressing shRNA, 1 μg packaging vector (pCMVdelta8.91) and 5 μg pMD-VSG-G for pseudotyped envelope. Lentiviral particles were collected after 48–72 h post transfection and titers were quantified in the supernatants as the amounts of HIV-1 p24 antigen using the Innotest HIV antigen mAB kit (Innogenetics N.V. Gent, Belgium). Lentiviral transduced cells were selected in puromycin-containing medium.

### Integration assay (Alu-LTR)

HIV-1 integration was examined in infected cells by isolating genomic DNA from 1×10^6^ cells using the DNeasy Tissue Kit (Qiagen). Alu-LTR sequences were amplified from 100–300 ng total genomic DNA in a first round PCR using LM667 and Alu1 primers, followed by a second round real-time PCR using one fifth of the first amplicon as a template together with the l-specific primer λT, the internal LTR primer LR and the probe ZXF-P^81^. Real-time PCR amplifications were performed on an AbiPrism 7000 machine, using the TaqMan technology (Applied Biosystem). Results were normalized to the amount of extracted cellular DNA of the single-copy Lamin B2 gene, quantified by real-time PCR.

### Luciferase assay

For the gene-silencing experiments, Pin1, LEDGF/p75, and TRIM33 depleted cells were infected with pseudotyped VSV-G pNL4.3.Luc.E-R- HIV-1 virus. Luciferase assays were performed after 48 h of infection with a Luciferase Assay kit (Promega). To normalize luciferase activity, total protein concentrations were determined in whole cell extracts of infected cells with the Bradford reagent (BioRad), according to manufacturer’s protocol).

### In vitro binding assays

In vitro translated S^35^-TRIM33 was produced using TNT-SP6 rabbit reticulocyte lysate (Promega) and pCS2-TRIM33 as a template. Full length GST-IN and its truncated variants (NTD, CCD, and CTD) purifications and in vitro binding were performed using standard procedures^82^. To remove contaminant bacterial nucleic acids, recombinant proteins were pretreated with nucleases (0.25 unit/µl Dnase I and 0.2 µg/µl RNase) for 1 h at 25 °C in 50 mM Tris HCl, pH 8.0, 5 mM MgCl2, 2.5 mM CaCl2, 100 mM NaCl, 5% glycerol, 1 mM DTT. Subsequently, GST fusion proteins immobilized on agarose beads were washed and resuspended in NETN buffer (20 mM Tris HCl, pH7.5, 100 mM NaCl, 1 mM EDTA, 0.5% NP-40, 1 mM DTT, 1 mM phenylmethylsulfonylfluoride). In vitro binding between GST-IN and GST-IN truncated variants were performed by incubating 2 µg of recombinant with 400 cpm of in vitro translated TRIM33 in a NETN buffer supplemented with 200 µg/ml ethidium bromide for 1 h at 4 °C on a rotating wheel, following five washes in NETN buffer and once wash with 10 mM Tris HCl pH 8.0/100 mM NaCl. Finally bound proteins were separated by electrophoresis in an SDS 10% polyacrylamide gel, analyzed by phosphoimaging (Cyclone) and stained with Coomassie Brilliant blue dye.

### RNA extraction and real-time PCR

Total mRNA was isolated from HeLa cells 72 h after siRNA transfection using a standard Trizol RNA extraction protocol. The RNA obtained (1 μg) was reverse-transcribed using MLV-RT (Invitrogen) with random hexameric primers (10 μM) in a 20-μl reaction following the manufacturer’s instructions. Quantification of VBP1 and TRIM33 gene expressions was performed by quantitative Real-Rime PCR using the forward and reverse primers. Expression of the housekeeping gene GAPDH was used for normalization. Amplifications were performed using a BIORAD CFX96 machine using GoTaq qPCR Master Mix (Promega). The Real-Time qPCR program was programmed with a melting curve dissociation protocol (from 60 °C to 95 °C), according to the manufacturer’s instructions.

### Primers and probes

The following oligodeoxynucletide primers and probes were used in this study.

Alu15′-TCCCAGCTACTGGGGAGGCTGAGG-3′

LM6675′-ATGCCACGTAAGCGAAACTCTGGCTAACTAGGGAACCCACTG-3′

LR5′-TCCACACTGACTAAAAGGGTCTGA-3′

λT5′-ATGCCACGTAAGCGAAACT-3′

ZXF-P (probe)5′-TGTGACTCTGGTAACTAGAGATCCCTCAGACCC-3′

B13 fwd5′-CCCCAGGGAGTAGGTTGTGA-3′

B13 rev5′-TGTTATTTGAGAAAAGCCCAAAGAC-3′

VBP1 fwd5′-TTGTGGCAAAGGAGAAATGGC-3′

VBP1 rev5′-CTCATTCCCAGGCTGTTTCATG-3′

TRIM33 fwd5′-AGCTTGACACCACCTCTCTC-3′

TRIM33 rev5′-CATGAGGCTTCGAATTGGGG-3′

## Supplementary information

Supplementary Information

## Data Availability

The authors declare that all data supporting the findings of this study are available within the paper and its supplementary information files. Primary raw data related to the high throughput screening results are available in Supplementary Table 1. Source data for blottings are shown in Supplementary Fig. 8.
