# Supplementary material for: Cellular TRIM33 restrains HIV-1 infection by targeting viral integrase for proteasomal degradation
Source: Nat Commun. 2019 Feb 25;10:926. doi: 10.1038/s41467-019-08810-0 (PMC6389893; doi:10.1038/s41467-019-08810-0)
Supplement: Supplementary file 1 — Supplementary Information [file 41467_2019_8810_MOESM1_ESM.pdf]

Ms. Ref. No.: NCOMMS-18-06181

Cellular TRIM33 restrains HIV-1 infection by targeting viral integrase for proteasomal degradation

Hashim Ali[1,5], Miguel Mano[1,#], Luca Braga[1,5], Asma Naseem[2], Bruna Marini[1,##], Diem My Vu[1], Chiara Collesi[1,4], Germana Meroni[3], Marina Lusic[1,## #] and Mauro Giacca[1,4,5]

[1]Molecular Medicine Laboratory, International Centre for Genetic Engineering and Biotechnology (ICGEB), Padriciano 99, 34149 Trieste, Italy

[2]Cellular Immunology Laboratory, International Centre for Genetic Engineering and Biotechnology (ICGEB), Padriciano 99, 34149 Trieste, Italy

[3] Department of Life Sciences, University of Trieste, 34127 Trieste, Italy

[4] Department of Medical, Surgical and Health Sciences, University of Trieste, 34127 Trieste, Italy

[5] Department of Cardiovascular Medicine & Sciences, King's College London, The James Black Centre, 125 Coldharbour Lane, London SE5 9N, UK

SUPPLEMENTARY FIGURES AND TABLES

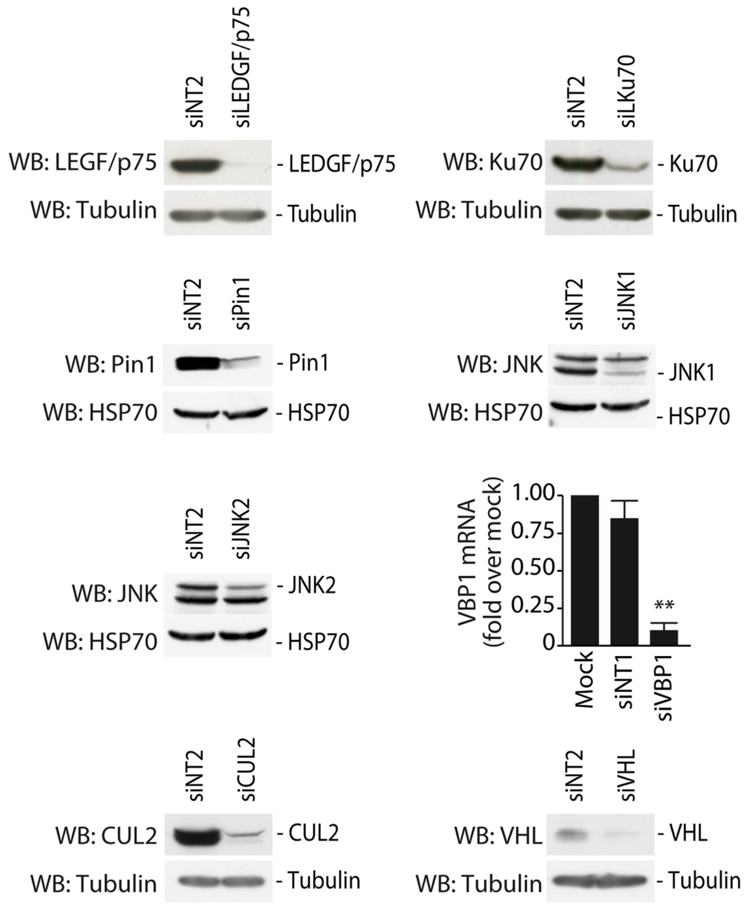

Supplementary Figure 1. Efficient knock-down of selected cellular proteins after RNAi. Amount of LEDGF/p75, Ku70, Pin1, JNK1, JNK2, Cul2, VHL by immunoblotting and of VBP1 mRNA by real-time RT-PCR after cells treatment with the respective siRNAs.

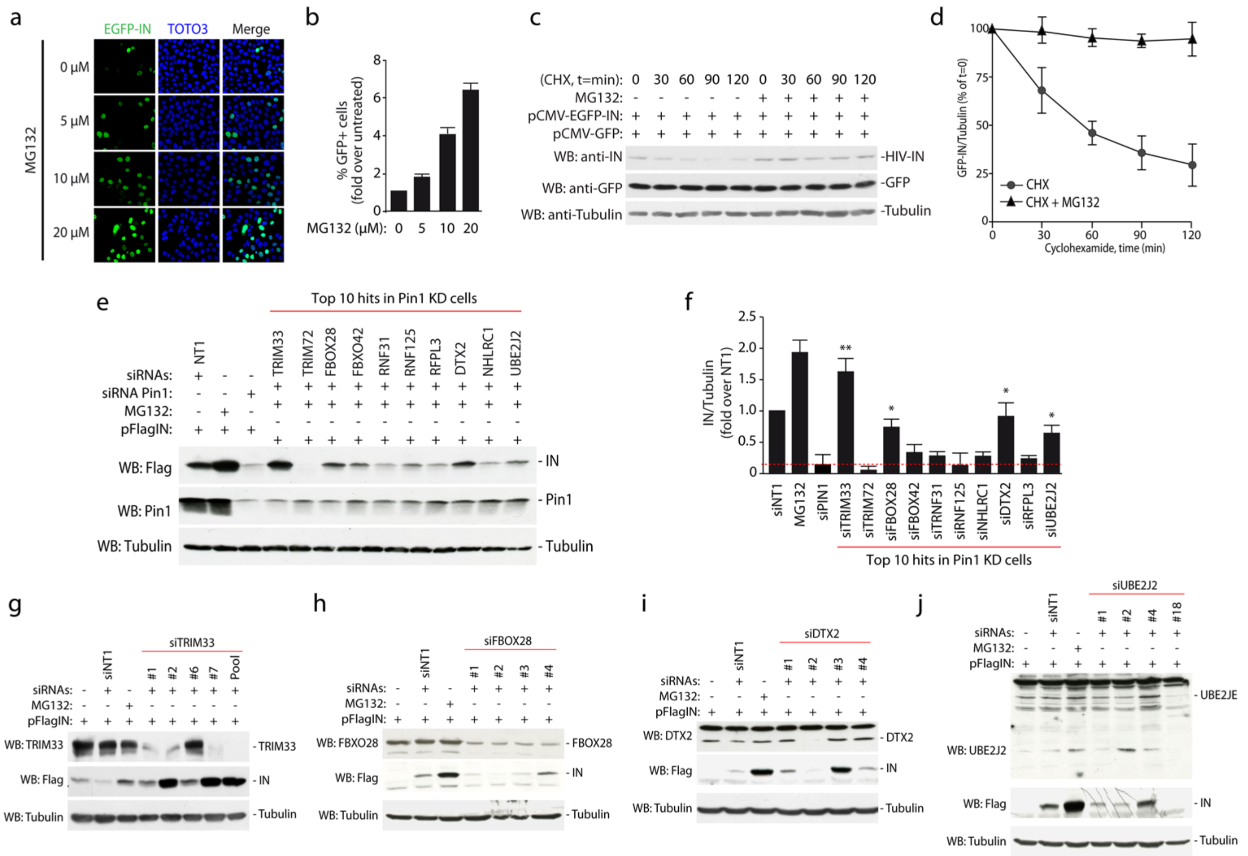

Supplementary Figure 2. Cellular TRIM33 E3 ligase determines degradation of HIV-1 integrase

a. Fusion to the EGFP protein does not interfere with the dynamics of HIV-1 IN degradation. HeLa cells were transfected with a plasmid expressing EGFP-IN and, after 48 hr, treated with the proteasome inhibitor MG132 for 5 hr at the indicated concentrations. Cells were then washed with 1XPBS; TOTO3 was used to stain nuclei and IN levels were examined by high content microscopy assessing the number of GFP+ cells.

b. Percentage of GFP+ cells, a surrogate for IN stability, at the indicated concentrations of MG132.

c. Measurement of EGFP-IN stability. EGFP-IN-expressing HeLa cells were treated with CHX (30 µg/ml) with or without MG132, followed by protein detection at different time points using an anti-IN antibody. Tubulin levels served as a loading control and GFP levels as a transfection and specificity control.

d. Quantification of EGFP-IN protein levels in the presence and absence of MG132 in CHX-treated cells at the indicated time points. Data are mean±SEM; n=3 independent experiments.

e. Representative immunoblot showing the levels of HIV-IN after knock-down of the top 10 E3 ligases from the screening together with an anti-Pin1 siRNA. HeLa cells were transfected with siRNAs against the indicated factors together with an siRNA against Pin1, followed by transfection of Flag-IN. Forty-eight hr later the levels of IN were assessed by anti-Flag immunoblotting. Tubulin served as a loading control.

f. Quantification of the levels of HIV-1 IN after knock-down of the top 10 E3 ligases identified by the screening together with the anti-Pin1 siRNA. Experiments were performed as in panel e. IN levels are expressed after normalization for tubulin and as fold over siNT1. Data are mean±SEM; n=3 independent experiments; **P*<0.05; ***P* <0.01; one-way ANOVA.

g. Effect of anti-TRIM33 siRNAs. The immunoblots show the levels of endogenous TRIM33 and transfected Flag-IN in HeLa cells treated with anti-TRIM33 siRNA pool (Pool), as used for the screening, its deconvoluted siRNAs (#1, #2, #6 and #7), or non-targeting siRNA-1 (siNT1). MG132 was used to assess the effect of inhibiting proteasome-mediated protein degradation.

h-j. Same as in panel g for FBOX28, DTX2 and UBE2J2 respectively.

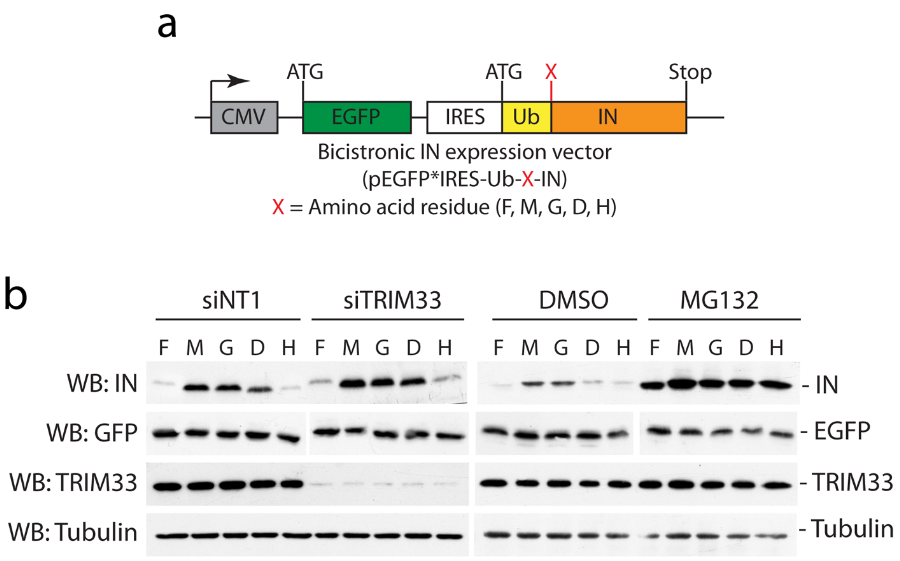

Supplementary Figure 3. TRIM33 is not involved in integrase degradation through the N-end rule pathway

a. Schematic representation of a bicistronic expression vector in which IN is expressed from as a fusion to an ubiquitin monomeric moiety immediately adjacent to its N-terminal amino acid [3]. The Ub-X-IN fusion protein is then co-translationally cleaved by the cell at the Ub-protein junction, thus producing IN with a desired residue ("X") at the N terminus of the protein [82].

b. Silencing of TRIM33 increases protein levels of IN N-terminal variants. HeLa cells were transfected with an siRNAs against TRIM33, followed by transfection of bicistroninc plasmids expressing IN carrying a different N-terminal residue (F: phenylalanine, M: methionine, G: glycine, D: aspartic acid, H: histidine). The levels of IN were assessed 48 hr later by anti-Flag immunoblotting. Tubulin served as a loading control and EGFP levels as transfection control. The results shown is representative of three different experiments.

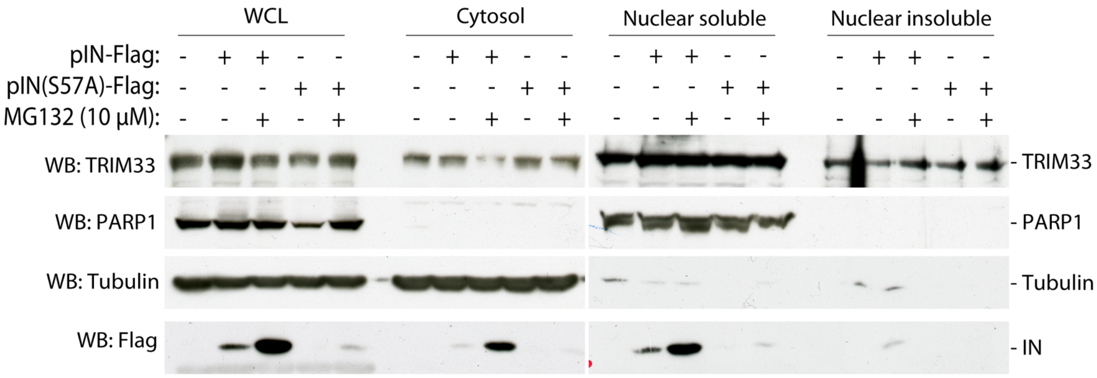

Supplementary Figure 4. TRIM33 is localized in both nucleus and cytoplasm.

Subcellular fractionation of HeLa cells transfected with wild type and S57A mutant in the presence or absence of proteasome inhibitor. Fractionated proteins were resolved on 10% SDS-PAGE gels before western blotting, Flag-IN and TRIM33 subcellular distribution was examined by indicated antibodies. Subcellular markers were Tubulin for the cytosolic fraction and PARP1 for the nuclear fraction.

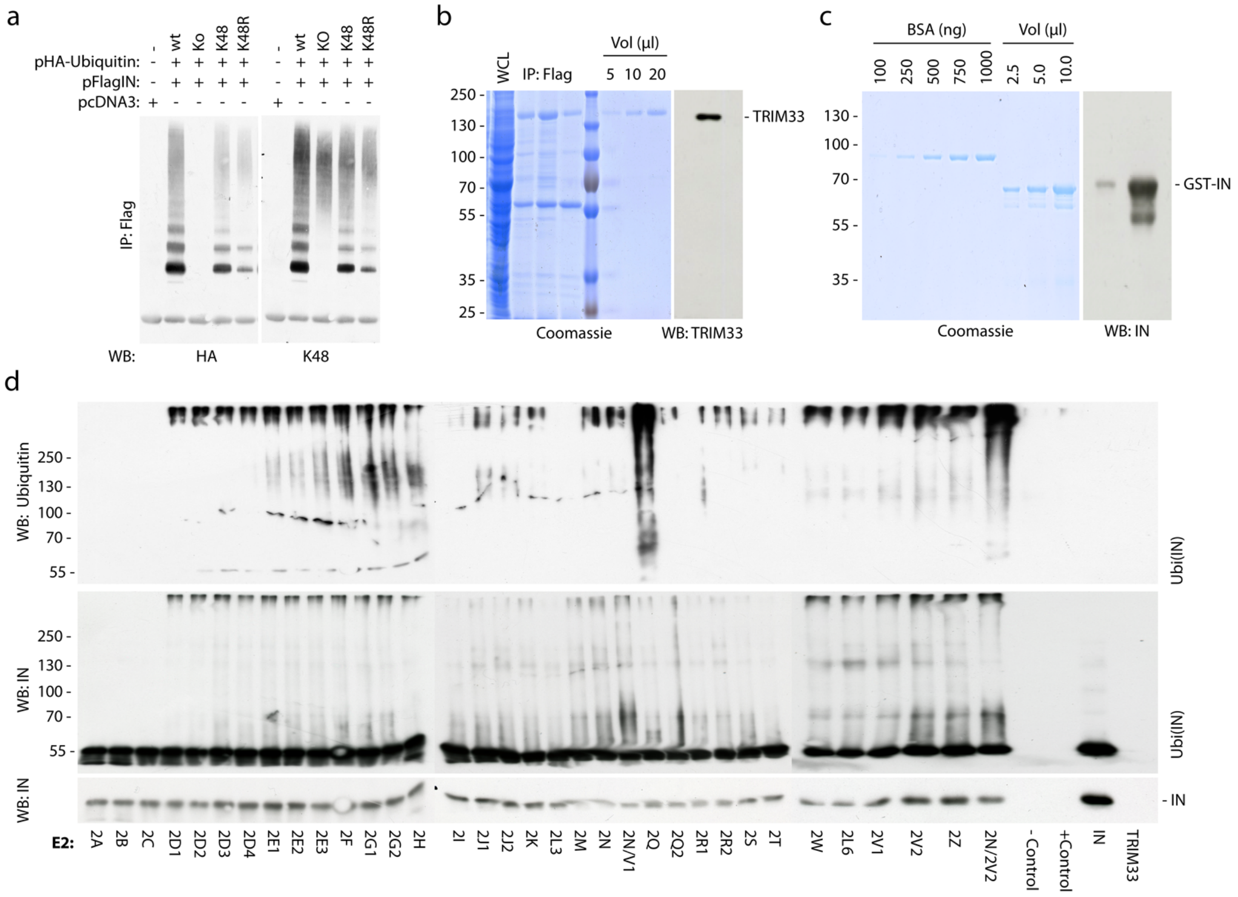

Supplementary Figure 5. Screening for the effect of E2-conjugating enzymes in TRIM33-dependent HIV-1 integrase poly-ubiquitination

a.. HIV-1 IN is mainly degraded via Lys48-linked polyubiquitination. HEK293T cells were cotransfected with FlagIN and HA- tagged ubiquitin or its K48, K48R and KO variants. K48 contains arginine substitutions on all the lysine residues except the one at position 48, it is thus expected to promote the proteasome-linked G76-K48 ubiquitin linkage. K48R contains a single lysine to arginine mutation at position 48 and is thus expected to disrupt the G76-K48 ubiquitin chain assembly. The KO ubiquitin mutant is a lysine-less ubiquitin only capable of mediating monoubiquitination. The ubiquitination profile of IN was assessed after immunoprecipitation with anti-Flag M2 beads, with, anti-HA and anti-K48 antibodies, as indicated.

b. Expression and purification of cellular TRIM33 from mammalian cells. HEK293T cells were transfected with plasmids expressing Flag-TRIM33 and immunoprecipitated with anti-Flag-M2 beads and eluted with Flag peptide. Purified TRIM33 was resolved on 10% SDS-PAGE, visualized using Coomassie Blue staining (left panel), and control by immunoblotting with anti-TRIM33 antibody (right panel).

c. Recombinant GST-IN purified from E. coli was resolved on 10% SDS-PAGE, visualized using Coomassie Blue staining (left panel), and monitored by immunoblotting with anti-IN antibody (right panel).

d. E2-conjugating enzyme screening by in vitro ubiquitination; Recombinant E1, different E2s as indicated and Ubiquitin were incubated with purified TRIM33 and IN in the presence of ATP and Mg^{2+} at 30°C for 90 min. Ubiquitination reactions were resolved on 8% SDS-PAGE, immunoblotted and visualized using an anti-ubiquitin antibody (shown in the upper panel) and anti-IN antibody (lower panel), Molecular weight (MW) markers are shown in kDa on the left side.

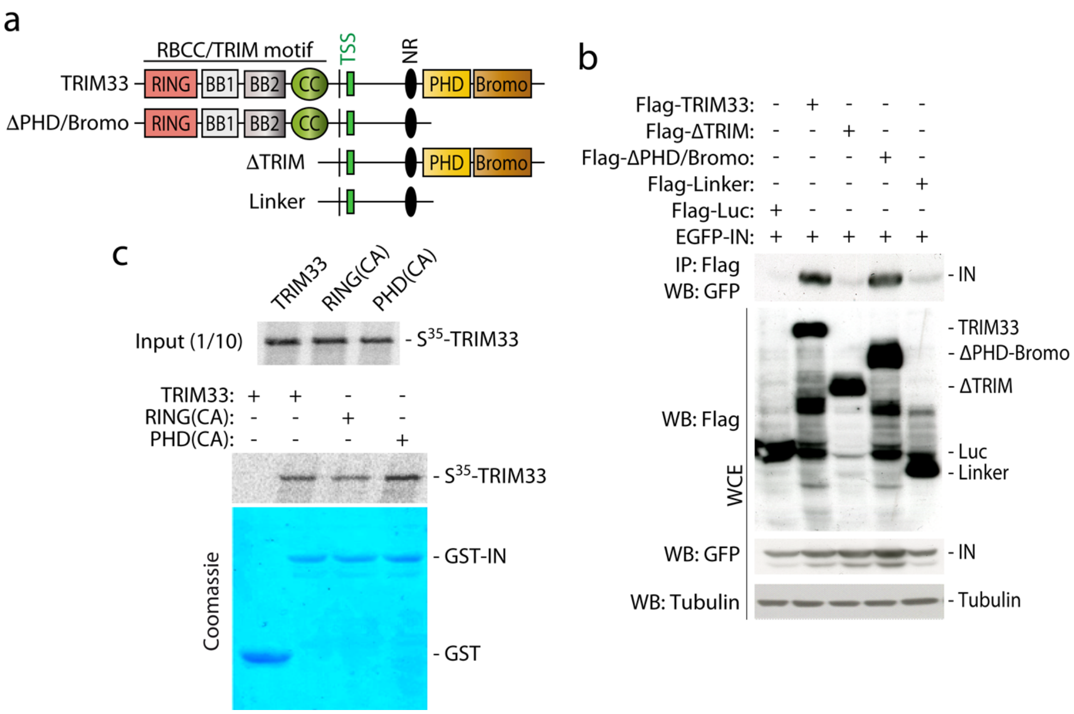

Supplementary Figure 6. TRIM33 inhibits viral replication by promoting degradation of viral integrase via poly-ubiquitination

a. Schematic representation of Flag-tagged TRIM33 and its deletion mutants.

b. The TRIM domain of TRIM33 is essential for binding HIV-1 IN. HEK 293T cells transfected with plasmids encoding Flag-TRIM33 and its truncated variants or Flag-luciferase together with EGFP-IN were immunoprecipitated with anti-Flag M2 beads and immunoblotted with an anti-GFP antibody. Whole cell extracts (WCE) were run on an SDS-PAGE gel and immunoblotted with anti-GFP, anti-Flag and anti-Tubulin antibodies to verify the amounts of expressed/loaded proteins.

c. Point mutations in TRIM33 RING and PHD domains do not alter binding to IN. In vitro translated, S^{35}-labeled TRIM33 and its mutants RING(C125A/C128A) and PHD(C901A/C902A/C905A) - RING(CA) and PHD(CA) respectively - were incubated with recombinant GST-IN or GST; binding was analyzed by phosphoimaging as in Fig. 4c. The top part of the figure shows the input levels of the three in vitro translated proteins used for the binding assays.

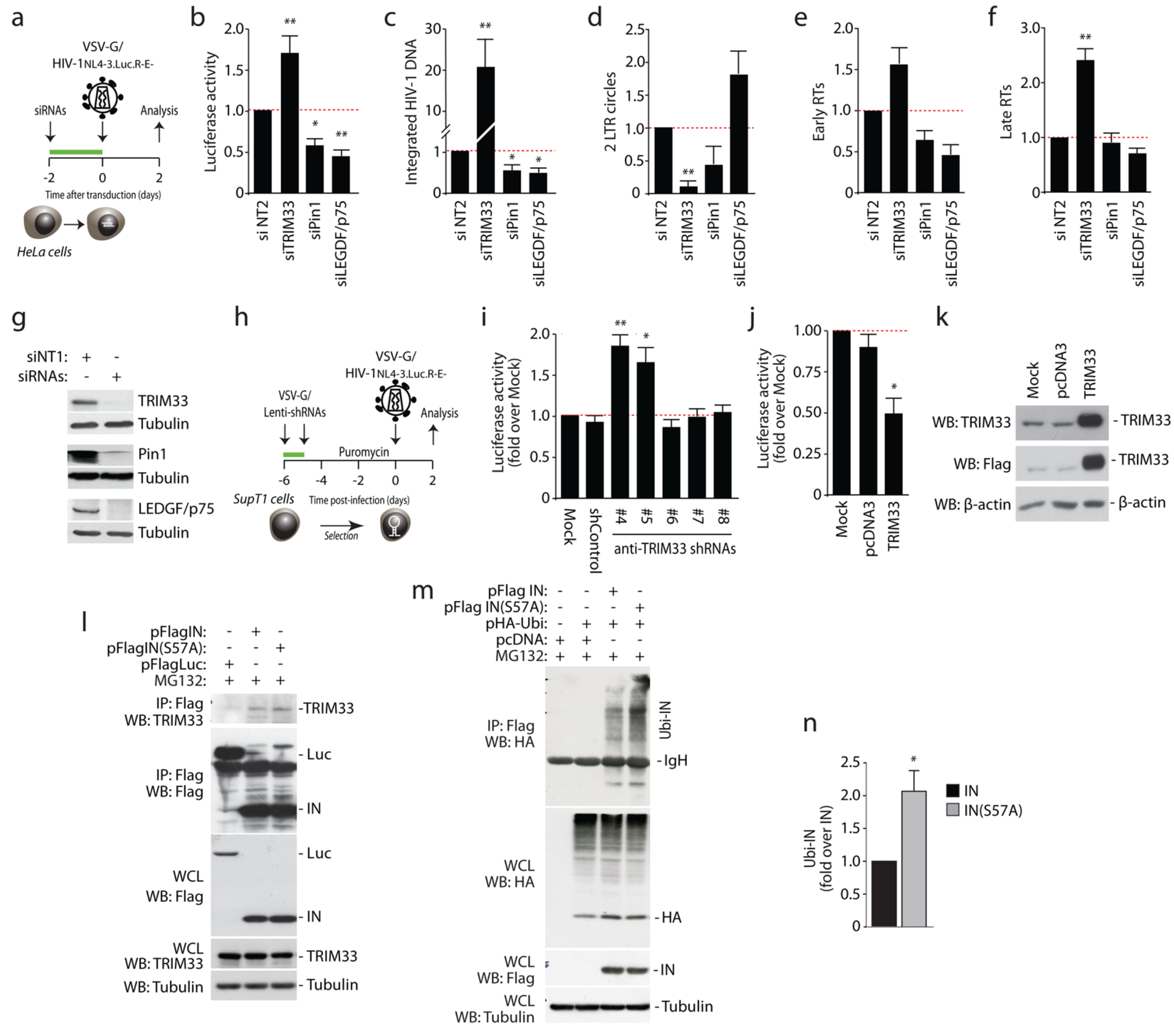

Supplementary Figure 7. TRIM33 knock down enhances viral infection

a. Transient silencing of TRIM33 enhances HIV-1 infection. HeLa cells were first transfected with siRNAs against TRIM33, Pin1, LEDGF/p75 or a control non-targeting siRNA (siNT2) and then infected with VSV-G-pseudotyped HIV-1$_{NL4-3.Luc.R-E-}$ molecular clone, followed by analysis of luciferase and of levels of DNA integration.

b. Results of luciferase assays after knockdown of the indicated factors. Data are mean±SEM; n=3 independent experiments; **P*<0.05; ***P* <0.01; one-way ANOVA.

c-f. Levels of integrated DNA (c), 2 LTR circles (d), early and late reverse transcripts (RT; e and f respectively) after knockdown of the indicated factors. DNA quantification results were normalized over the levels of the B13 single-copy cellular gene [77]. Data are mean±SEM; n=3 or more independent experiments; **P*<0.05; ***P* <0.01; one-way ANOVA

g. Western blot showing protein levels for Pin1, LEDGF/p75 and TRIM33 in infected cells transfected with the respective siRNAs.

h. Scheme of procedure to assess the effect of stable knockdown of TRIM33 in-single round HIV-1 infection. SupT1 cells were transduced with one of five lentiviral vectors expressing different shRNAs against TRIM33 (#4,

#5, #6, #7, and #8) or a control, non-targeting shRNA. Forty-eight hr after infection, cells were grown in puromycin-containing medium to selected transduced cells. After four additional days, cells with infected with VSV-G-pseudotyped HIV-$1_{NL4\text{-}3.Luc.R\text{-}E\text{-}}$ and analysed for luciferase activity at day 2 after infection.

i. Luciferase activity in SupT1 cells transduced with lentiviral vectors expressing the indicated shRNAs. Data are mean±SEM; n=3 independent experiments; *P<0.05; **P <0.01; one-way ANOVA.

j. Luciferase activity in cells transfected with an expression vector for TRIM33 and infected with VSVG-/ HIV-$1_{NL4\text{-}3.Luc.R\text{-}E\text{-}}$.

k. Western blotting showing the levels of expression of TRIM33 in transfected cells.

l. Endogenous TRIM33 interacts with both wild type IN and the IN(S57A) mutant in vivo. In the upper picture, HEK293T cells were transfected with wild type IN-, mutant IN(S57A)-, and luciferase expressing plasmids; lysates were then immunoprecipitated with anti-Flag M2 beads and immunoblotted with anti-TRIM33 antibody. The lower three pictures show control immunoblottings with the indicated antibodies to monitored the expression levels of the indicated proteins in whole cell lysates (WCL). The blot is representative of at least three experiments showing consistent results.

m. Representative immunoblotting showing the levels of poly-ubiquitination of wild type IN and the IN(S57A) mutant. HEK293T cells were transfected with combinations of HA-ubiquitin, Flag-IN or Flag-IN(S57A). Cells were harvested after 5 hr treatment with MG132 and immunoprecipitated with anti-Flag M2 beads. Ubiquitin-conjugated IN (Ubi-IN) was detected by immunoblotting with anti-HA antibody (upper picture). The lower pictures show the expression of the transfected proteins in whole cell lysates (WCL) after immunoblotting with the indicated antibodies.

n. Quantification of the levels of ubiquitination of IN and IN(S57A). Experiments were performed as in panel e. Data are mean±SEM; n=3 independent experiments; *P<0.05; t-test.

Supplementary Figure 8/1 - 8/7 (next 7 pages). Original source blots for the panels shown in the main manuscript and supplementary figures. The portion of the image that is displayed is boxed with a dotted red line.

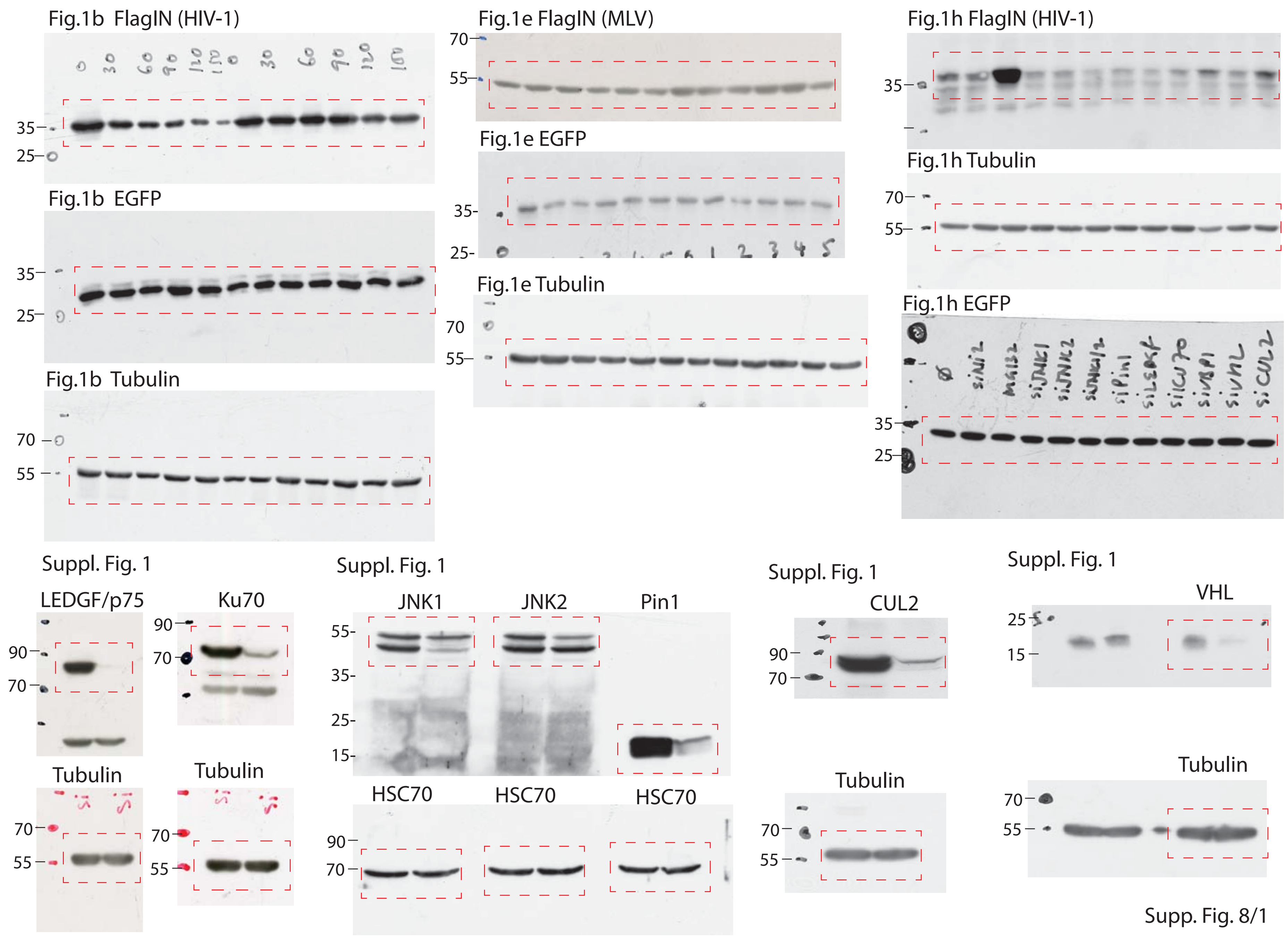
Fig.1b FlagIN (HIV-1)
35
25
Fig.1b EGFP
35
25
Fig.1b Tubulin
70
55
Fig.1e FlagIN (MLV)
70
55
Fig.1e EGFP
35
25
Fig.1e Tubulin
70
55
Fig.1h FlagIN (HIV-1)
35
Fig.1h Tubulin
70
55
Fig.1h EGFP
35
25
Suppl. Fig. 1
LEDGF/p75
90
70
Ku70
90
70
Tubulin
70
55
Tubulin
70
55
Suppl. Fig. 1
JNK1
JNK2
Pin1
55
35
25
15
HSC70
HSC70
HSC70
90
70
Suppl. Fig. 1
CUL2
90
70
Tubulin
70
55
Suppl. Fig. 1
VHL
25
15
Tubulin
70
55

Supp. Fig. 8/1

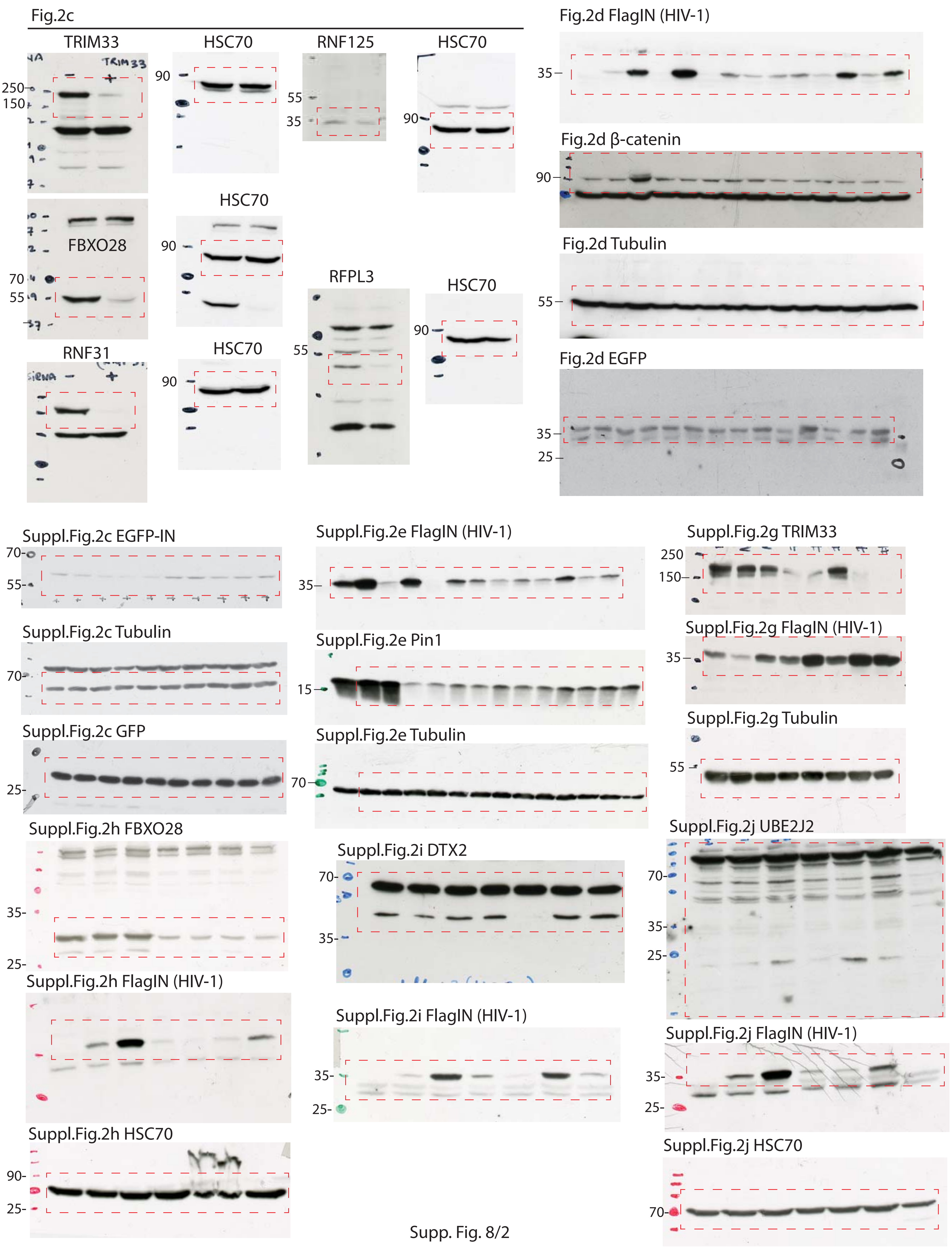
Fig.2c
TRIM33
HSC70
RNF125
HSC70
250
150
90
55
35
90
FBXO28
HSC70
70
55
90
RFPL3
HSC70
55
90
RNF31
HSC70
90
Fig.2d FlagIN (HIV-1)
35
Fig.2d β-catenin
90
Fig.2d Tubulin
55
Fig.2d EGFP
35
25
Suppl.Fig.2c EGFP-IN
70
55
Suppl.Fig.2c Tubulin
70
Suppl.Fig.2c GFP
25
Suppl.Fig.2e FlagIN (HIV-1)
35
Suppl.Fig.2e Pin1
15
Suppl.Fig.2e Tubulin
70
Suppl.Fig.2g TRIM33
250
150
Suppl.Fig.2g FlagIN (HIV-1)
35
Suppl.Fig.2g Tubulin
55
Suppl.Fig.2h FBXO28
35
25
Suppl.Fig.2h FlagIN (HIV-1)
Suppl.Fig.2h HSC70
90
25
Suppl.Fig.2i DTX2
70
35
Suppl.Fig.2i FlagIN (HIV-1)
35
25
Suppl.Fig.2j UBE2J2
70
35
25
Suppl.Fig.2j FlagIN (HIV-1)
35
25
Suppl.Fig.2j HSC70
70

Supp. Fig. 8/2

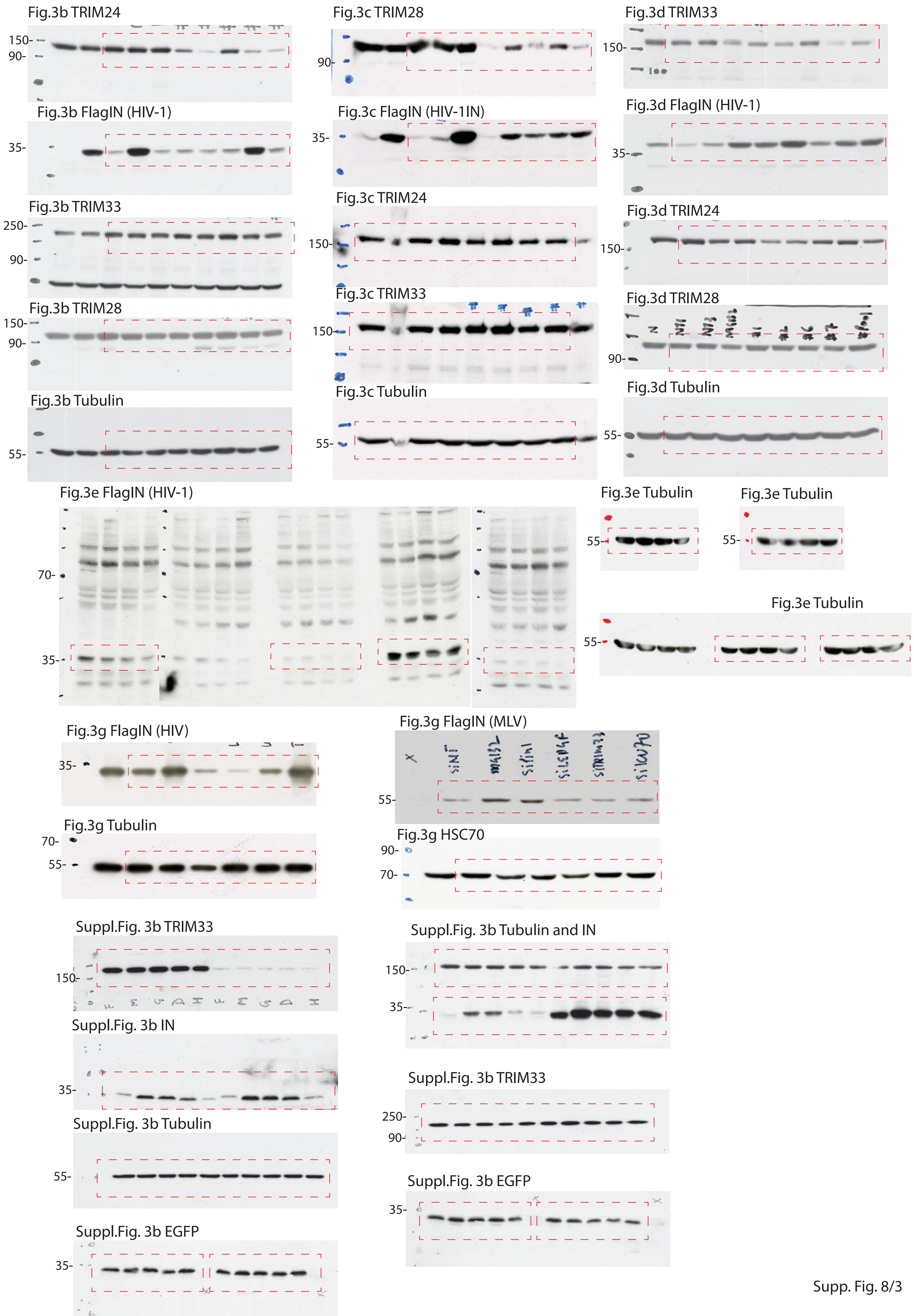
Fig.3b TRIM24
150-
90-
Fig.3b FlagIN (HIV-1)
35-
Fig.3b TRIM33
250-
90-
Fig.3b TRIM28
150-
90-
Fig.3b Tubulin
55-
Fig.3c TRIM28
90-
Fig.3c FlagIN (HIV-1IN)
35-
Fig.3c TRIM24
150-
Fig.3c TRIM33
150-
Fig.3c Tubulin
55-
Fig.3d TRIM33
150-
Fig.3d FlagIN (HIV-1)
35-
Fig.3d TRIM24
150-
Fig.3d TRIM28
90-
Fig.3d Tubulin
55-
Fig.3e FlagIN (HIV-1)
70-
35-
Fig.3e Tubulin
55-
Fig.3e Tubulin
55-
Fig.3e Tubulin
55-
Fig.3g FlagIN (HIV)
35-
Fig.3g FlagIN (MLV)
55-
Fig.3g Tubulin
70-
55-
Fig.3g HSC70
90-
70-
Suppl.Fig. 3b TRIM33
150-
Suppl.Fig. 3b Tubulin and IN
150-
35-
Suppl.Fig. 3b IN
35-
Suppl.Fig. 3b TRIM33
250-
90-
Suppl.Fig. 3b Tubulin
55-
Suppl.Fig. 3b EGFP
35-
Suppl.Fig. 3b EGFP
35-

Supp. Fig. 8/3

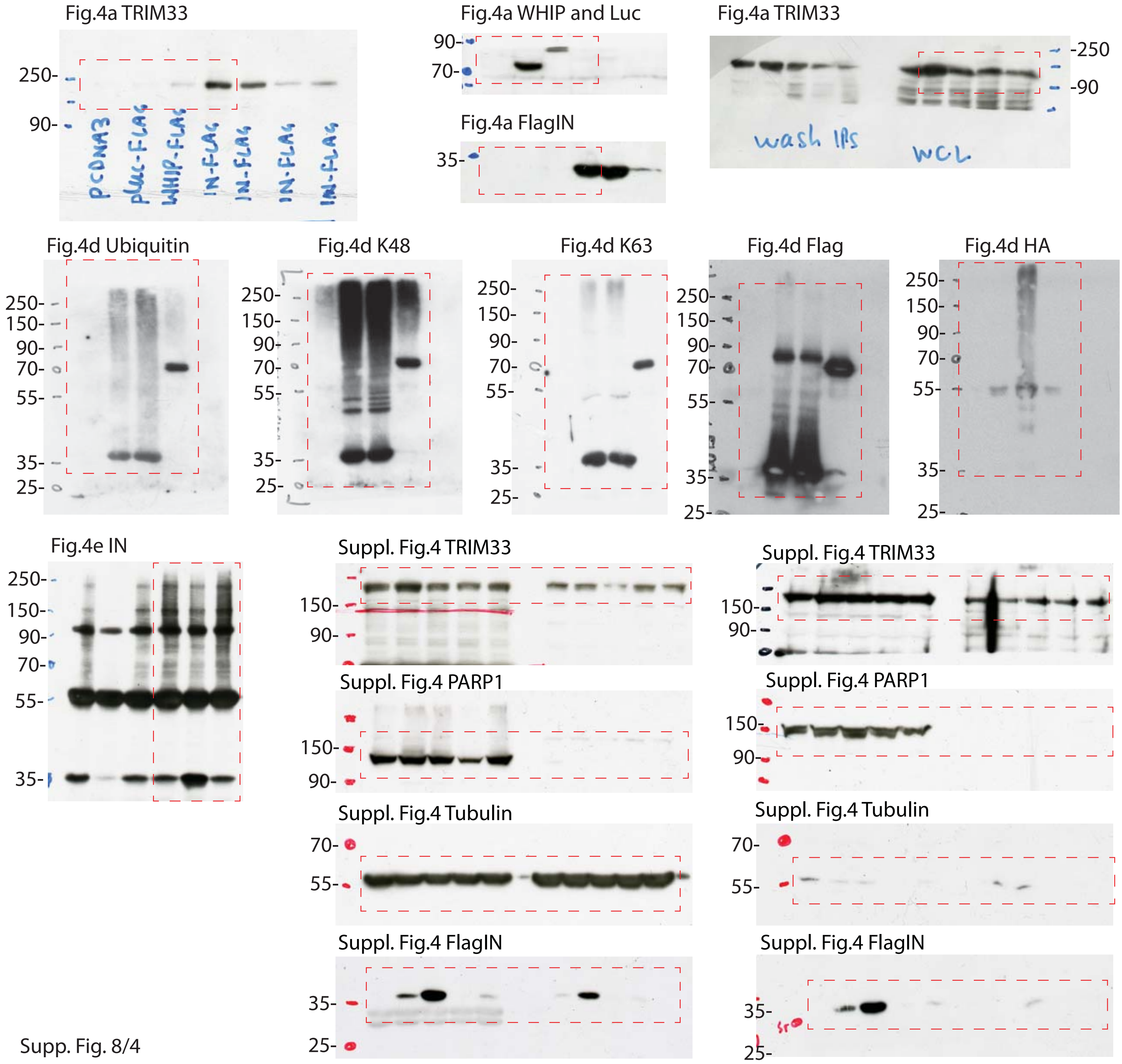
Fig.4a TRIM33
250-
90-
pCDNA3
pLuc-FLAG
WHIP-FLAG
IN-FLAG
IN-FLAG
IN-FLAG
IN-FLAG
Fig.4a WHIP and Luc
90-
70-
Fig.4a FlagIN
35-
Fig.4a TRIM33
-250
-90
wash IPs
WCL
Fig.4d Ubiquitin
250-
150-
90-
70-
55-
35-
25-
Fig.4d K48
250-
150-
90-
70-
55-
35-
25-
Fig.4d K63
250-
150-
90-
70-
55-
35-
25-
Fig.4d Flag
250-
150-
90-
70-
55-
35-
25-
Fig.4d HA
250-
150-
90-
70-
55-
35-
25-
Fig.4e IN
250-
150-
90-
70-
55-
35-
Suppl. Fig.4 TRIM33
150-
90-
Suppl. Fig.4 TRIM33
150-
90-
Suppl. Fig.4 PARP1
150-
90-
Suppl. Fig.4 PARP1
150-
90-
Suppl. Fig.4 Tubulin
70-
55-
Suppl. Fig.4 Tubulin
70-
55-
Suppl. Fig.4 FlagIN
35-
25-
Suppl. Fig.4 FlagIN
35-
25-

Supp. Fig. 8/4

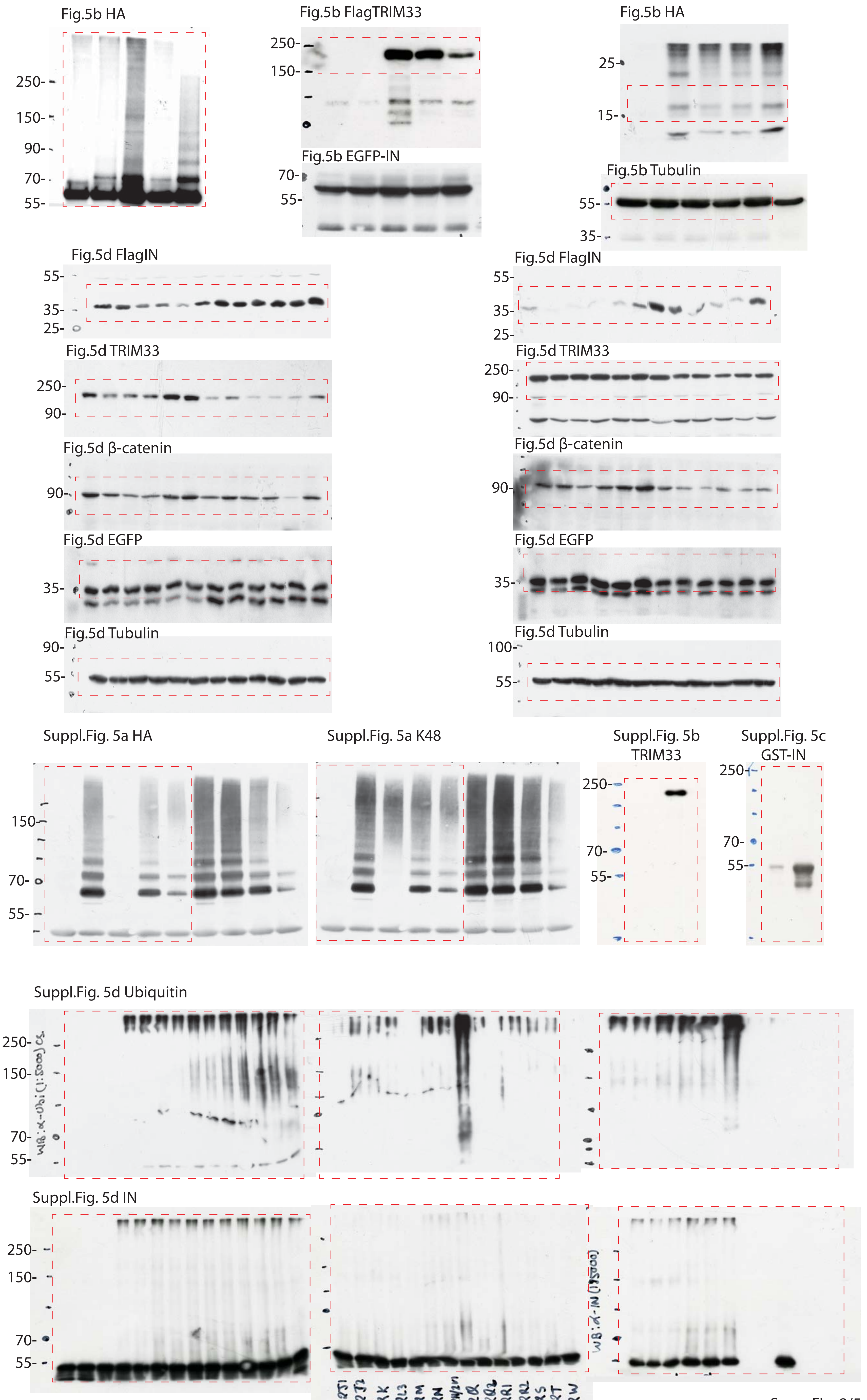

Fig.5b HA
Fig.5b FlagTRIM33
Fig.5b EGFP-IN
Fig.5b Tubulin
Fig.5d FlagIN
Fig.5d TRIM33
Fig.5d β-catenin
Fig.5d EGFP
Fig.5d Tubulin
Suppl.Fig. 5a HA
Suppl.Fig. 5a K48
Suppl.Fig. 5b TRIM33
Suppl.Fig. 5c GST-IN
Suppl.Fig. 5d Ubiquitin
Suppl.Fig. 5d IN

Supp. Fig. 8/5

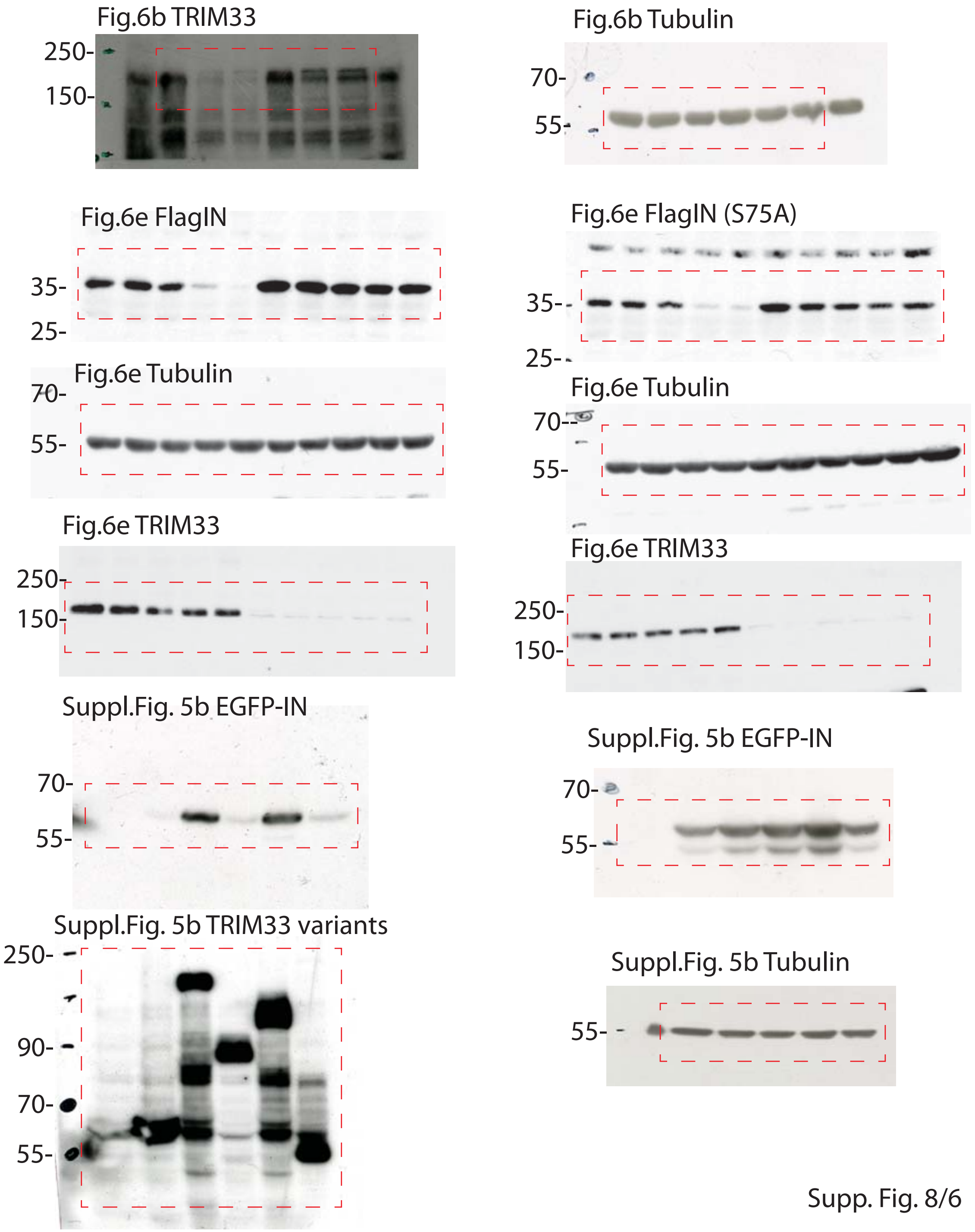
Fig.6b TRIM33
250-
150-
Fig.6b Tubulin
70-
55-
Fig.6e FlagIN
35-
25-
Fig.6e FlagIN (S75A)
35-
25-
Fig.6e Tubulin
70-
55-
Fig.6e Tubulin
70-
55-
Fig.6e TRIM33
250-
150-
Fig.6e TRIM33
250-
150-
Suppl.Fig. 5b EGFP-IN
70-
55-
Suppl.Fig. 5b EGFP-IN
70-
55-
Suppl.Fig. 5b TRIM33 variants
250-
90-
70-
55-
Suppl.Fig. 5b Tubulin
55-

Supp. Fig. 8/6

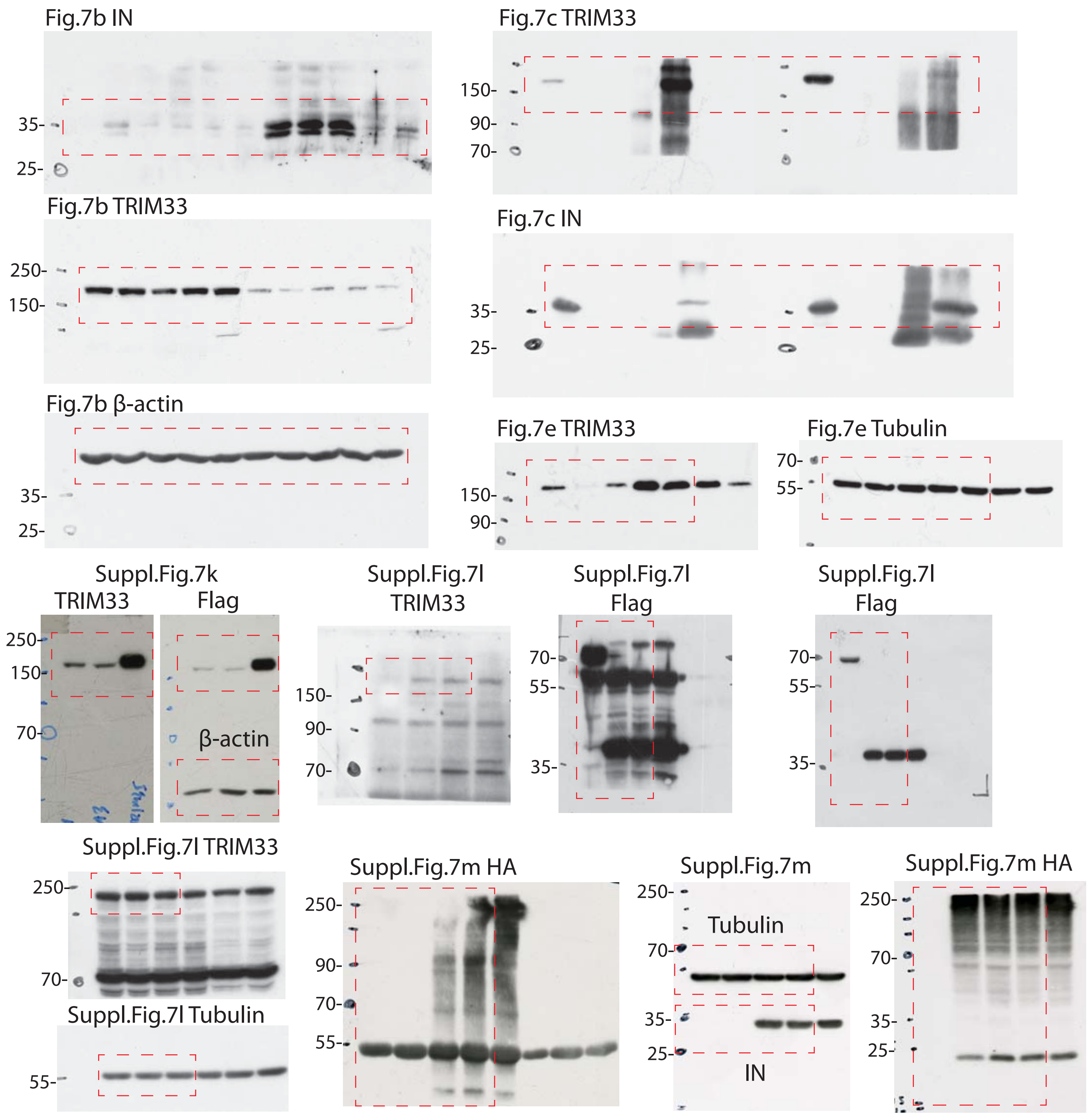
Fig.7b IN
35-
25-
Fig.7b TRIM33
250-
150-
Fig.7b β-actin
35-
25-
Fig.7c TRIM33
150-
90-
70-
Fig.7c IN
35-
25-
Fig.7e TRIM33
150-
90-
Fig.7e Tubulin
70-
55-
Suppl.Fig.7k
TRIM33
Flag
250-
150-
70-
β-actin
Suppl.Fig.7l
TRIM33
150-
90-
70-
Suppl.Fig.7l
Flag
70-
55-
35-
Suppl.Fig.7l
Flag
70-
55-
35-
Suppl.Fig.7l TRIM33
250-
70-
Suppl.Fig.7l Tubulin
55-
Suppl.Fig.7m HA
250-
90-
70-
55-
Suppl.Fig.7m
250-
Tubulin
70-
35-
25-
IN
Suppl.Fig.7m HA
250-
70-
35-
25-

Supp. Fig. 8/7

Suppl. Table 1. List of siRNA target genes and results of HTS. Color code is as in Fig. 2b

Gene Symbol	Entrez Gene ID	Log10 fold over Control (Replicate 1)	Log10 fold over Control (Replicate 2)
UBE2J2	118424	0.577921286	0.864647815
RFPL3	10738	0.734611194	0.772161971
FBXO28	23219	0.694426882	0.725664128
MG132		0.469522765	0.683302764
RNF19	25897	0.274082765	0.609413291
RNF31	55072	0.523513784	0.560505013
DTX2	113878	0.337581046	0.527672555
TRIM33	51592	0.441829251	0.475263283
RNF125	54941	0.338229725	0.462988985
FBXO42	54455	0.449923419	0.453957689
FBXO4	26272	0.185175697	0.444567977
KIAA1333	55632	0.234377305	0.443066689
NHLRC1	378884	0.470957645	0.418665647
ZNRF1	84937	0.337581046	0.40847164
TRIM65	201292	-0.139540228	0.39299892
DCUN1D2	55208	0.288855964	0.370880428
TRIM72	493829	0.463977899	0.367314167
MDM2	4193	0.364329758	0.3641193
RNF169	254225	0.113671666	0.360698851
RNF151	146310	0.056222778	0.34715802
LONRF3	79836	0.378442237	0.31156167
EDD1	51366	0.218445959	0.307699232
RKHD1	399664	0.407577501	0.301956009
UBE2G2	7327	-0.159465533	0.28569905
LRRC29	26231	0.187933105	0.272537546
UBE4A	9354	0.070863534	0.267309507
MARCH4	57574	0.198791203	0.252015905
FBXL11	22992	0.013214012	0.246533293
M96	22823	-0.427147326	0.244426314
LNX2	222484	-0.08386718	0.241512363
LOC642219	642219	0.09250941	0.240980583
UBE2I	7329	0.169210802	0.23481658
RNF128	79589	0.171119773	0.231837209
ASB6	140459	0.115299239	0.223606512
TSG101	7251	0.197896681	0.219152112
TRIML1	339976	0.337581046	0.197640804
TRIM15	89870	0.222272328	0.197640804
TRIM6-TRIM34	445372	0.066644167	0.191123353
FSD1L	83856	0.405361559	0.189628413
LOC390231	390231	0.053734002	0.189328862
RNF182	221687	0.203236381	0.182988672
FBXL16	146330	-0.340168767	0.179630198
PDC	5132	0.408956638	0.178402494
CUL1	8454	-0.029207248	0.164346609
UBE4B	10277	-0.159465533	0.160520151
FBXW5	54461	0.098179423	0.157628095
AKTIP	64400	0.313215501	0.156659679
PCGF2	7703	0.337905507	0.154391712
TRIM25	7706	-0.176091237	0.152112144
BIRC8	112401	0.115299239	0.152112144
TRIM62	55223	-0.225393995	0.14053004
LOC441061	441061	0.141564525	0.135470411
ZNF179	7732	-0.111268185	0.134451245
BAHD1	22893	0.004216442	0.132747516
MKRN3	7681	0.134886959	0.115331542
SOCS4	122809	0.130203139	0.112836831
RAD18	56852	-0.205676361	0.100510462
RAG1	5896	-0.194482559	0.091593807
RNF34	80196	-0.152387244	0.088958165
LOC51136	51136	-0.007119713	0.086686497
SPSB3	90864	-0.929736728	0.082490237

Gene Symbol	Entrez Gene ID	Log10 fold over Control (Replicate 1)	Log10 fold over Control (Replicate 2)
RNF7	9616	-0.020977513	0.077865877
STUB1	10273	-0.195589031	0.075924524
LOC728919	728919	0.349743246	0.072015588
PHF10	55274	0.199683886	0.071622825
RNF38	152006	-0.206811761	0.065288345
NSD1	64324	0.350374023	0.062086117
MYLIP	29116	-0.096910013	0.061282045
PRPF19	27339	0.378737572	0.061282045
WHSC1	7468	-0.438627029	0.058860102
RBX1	9978	-0.362363896	0.056017451
MARCH8	220972	0.031982969	0.05233528
RNF12	51132	0.168253161	0.049863169
TULP4	56995	0.15708548	0.049863169
WWP2	11060	-0.043800344	0.046960815
TRIM71	131405	-0.329482612	0.040675903
WDR71	80227	0.128630249	0.04025334
SPSB2	84727	-0.089037415	0.039407773
UBE2D1	7321	-0.194482559	0.034726292
FBXO18	84893	0.349743246	0.031721044
ARIH1	25820	-0.172925074	0.031721044
LOC643904	643904	-0.126117272	0.029127996
MIB2	142678	-0.176091237	0.027390792
FBXO31	79791	-0.010723846	0.026955506
RNF4	6047	0.238474412	0.025209572
LOC644006	644006	0.136952552	0.024334172
MKRN2	23609	-0.011448267	0.020813989
PCGF1	84759	0.116380909	0.020372057
SHPRH	257218	-0.06133385	0.01904314
TRIM68	55128	-0.803448565	0.016373044
RFWD3	55159	-0.017287639	0.015926567
BRPF1	7862	-0.048512447	0.012337288
TIP120A	55832	0.033946605	0.010531462
LNX1	84708	-0.209091504	0.010078936
CCNF	899	0.083281116	0.003232554
ASB17	127247	0.042354568	0.003232554
C1orf164	55182	-0.217166259	0.001388389
UBE2Q1	55585	-0.307432511	-0.000463727
NT1		*-0.341717039*	*-0.011268831*
AMFR	267	-0.2160035	-0.013175706
PHF11	51131	-0.326477132	-0.017497069
RNF41	10193	-0.049302784	-0.020402099
MARCH5	54708	-0.2823701	-0.021861864
DCUN1D1	54165	-0.089037415	-0.022349591
ASB15	142685	-0.254848979	-0.024305902
FBXW2	26190	-0.30600157	-0.025778871
LOC642678	642678	0.212425735	-0.028245068
LOC653192	653192	-0.206811761	-0.032719843
PDZRN4	29951	-0.494408894	-0.034221724
TRIM42	287015	-0.011448267	-0.034723492
HR	55806	-0.153391384	-0.037746428
UBE2Q2	92912	0.039139971	-0.040281684
RNF113A	7737	-0.166661101	-0.041300021
UBE2A	7319	0.184714422	-0.041300021
CUL4B	8450	-0.075384576	-0.042320656
TRIM47	91107	-0.244807065	-0.044882882
UNK	85451	-0.316118693	-0.047460315
UBE2V2	7336	0.098742339	-0.047460315
C6ORF49	29964	-0.298916657	-0.049533303
TRIM74	378108	0.094786166	-0.050573543
TRAF5	7188	-0.220673403	-0.05370925
RAB40B	10966	-0.45442138	-0.054234059
BIRC6	57448	0.054980173	-0.054759553
KRTAP5-9	3846	-0.028452617	-0.056339762

Gene Symbol	Entrez Gene ID	Log10 fold over Control (Replicate 1)	Log10 fold over Control (Replicate 2)
FBXO6	26270	-0.298916657	-0.057396454
SSA1	6737	-0.175033283	-0.058455674
HACE1	57531	-0.22184875	-0.060049413
C10ORF46	143384	-0.279672636	-0.060581995
RNF139	11236	-0.338625994	-0.061649072
FBXO46	23403	0.547184131	-0.06218357
TRIM45	80263	-0.436692548	-0.063254545
LOC644006	644006	-0.329482612	-0.064866075
BIRC2	329	-0.259958412	-0.070281259
FLJ10916	55258	-0.193378898	-0.072466422
FBXW10	10517	-0.6650234	-0.073563089
RSPRY1	89970	-0.609898089	-0.075764869
FBXL10	84678	-0.014358184	-0.076317037
DZIP3	9666	0.141564525	-0.077423535
UBE2B	7320	-0.128009607	-0.082437884
TRIM73	375593	-0.452415417	-0.082998607
UBOX5	22888	-0.234992105	-0.084122282
ARIH1	25820	-0.143452839	-0.084122282
UBE2W	55284	-0.128009607	-0.092643682
JARID1B	10765	0.04744894	-0.0932178
DTX3	196403	-0.334030288	-0.097257751
TRIM29	23650	0.264971215	-0.099583326
RNF186	54546	0.311150514	-0.105452283
TRIAD3	54476	-0.073707727	-0.109608215
FBXO47	494188	-0.329482612	-0.111401583
UBE2E3	10477	0.167293404	-0.115010691
KIAA1542	57661	0.073256323	-0.115010691
HUWE1	10075	-0.269047108	-0.125402463
ZFAND6	54469	-0.300324406	-0.128506809
BMI1	648	-0.118629268	-0.128506809
LOC200933	200933	-0.031479087	-0.129130327
FBXO7	25793	0.02202895	-0.130380112
TRIM3	10612	-0.39442347	-0.130380112
HECTD1	25831	-0.122357161	-0.131006327
PHF7	51533	-0.047723546	-0.134782875
ZSWIM2	151112	0.089071738	-0.135415475
TRIM22	10346	0.124408683	-0.136048998
FBXO15	201456	0.116380909	-0.138592491
HERC3	8916	-0.207950137	-0.138592491
RC3H1	149041	-0.39442347	-0.139869846
MID2	11043	-0.592975553	-0.141150908
PHF5A	84844	-0.223027285	-0.141792859
SYTL3	94120	0.059313826	-0.141792859
RNF113B	140432	-0.010000631	-0.142435821
LOC652591	652591	-0.346395351	-0.143079676
TRIM28	10155	-0.72068926	-0.147613768
RNF207	388591	-0.209091504	-0.148265351
UBE1	7317	-0.256120693	-0.148265351
WDR59	79726	-0.176091237	-0.151538133
UBE1DC1	79876	-0.230166535	-0.154174196
ZNRF2	223082	-0.079605168	-0.154835766
SOCS6	9306	-0.273001218	-0.154835766
CUL5	8065	-0.681665107	-0.157491983
RNF11	26994	-0.188992025	-0.15815863
NEURL	9148	-0.278330162	-0.15882624
ZNF185	7739	0.011841846	-0.159494877
UBE2R2	54926	-0.036826662	-0.162853719
RNF43	54894	0.192943605	-0.163528591
TRIM38	10475	-0.102238848	-0.164881552
ASB18	401036	-0.815747343	-0.165559585
EEA1	8411	-0.104927959	-0.166918898
ASB9	140462	-0.298916657	-0.167600186
PHF1	5252	-0.293330769	-0.17033588

Gene Symbol	Entrez Gene ID	Log10 fold over Control (Replicate 1)	Log10 fold over Control (Replicate 2)
AIRE	326	-0.612783926	-0.17033588
HERC2	8924	-0.290564581	-0.171022489
SYTL4	94121	0.176326583	-0.173088917
FLJ34154	283450	-0.415963155	-0.177949209
TRIM26	7726	0.119609847	-0.177949209
RAB40C	57799	-0.311753842	-0.18004894
TRIM46	80128	-0.090774578	-0.180751156
TRIM41	90933	-0.134698594	-0.181454443
FBXL3P	26223	-0.257396221	-0.186409739
FBXL7	23194	-0.097793615	-0.189266987
INTS12	57117	-0.226582277	-0.189984213
PHF19	26147	0.201019479	-0.189984213
DCUN1D5	84259	-0.123294141	-0.192865154
RUFY1	80230	0.186556589	-0.195038437
CUL3	8452	-0.167698832	-0.197953146
MSL2L1	55167	0.007699141	-0.200152105
TRIM6	117854	-0.505573918	-0.200887618
DPF3	8110	-0.224209028	-0.202362255
SPSB1	80176	-0.097793615	-0.202362255
FBXL18	80028	-0.808871612	-0.203101454
SPRYD5	84767	0.065431066	-0.203841984
TRIM54	57159	-0.128958934	-0.205326773
RNF5	6048	-0.332509036	-0.20607104
UBE2E2	7325	-0.088171379	-0.207563482
PHF6	84295	-0.685071321	-0.209811701
MLL3	58508	-0.430940277	-0.210563771
BARD1	580	-0.517033567	-0.215864692
FBXL4	26235	-0.403268789	-0.215864692
PDZRN3	23024	-0.460495466	-0.216627327
DKFZP547C195	257160	-0.514717205	-0.218923157
UBR1	197131	-0.152387244	-0.218923157
RC3H2	54542	-0.113096778	-0.219691187
SOCS7	30837	-0.483523726	-0.222003241
ARIH2	10425	-0.380635111	-0.225884376
TRIM75	391714	-0.397940009	-0.225884376
KIAA0804	23355	-0.290564581	-0.225884376
TRIM5	85363	-0.355905831	-0.225884376
MYCBP2	23077	-0.102238848	-0.23295907
TRIM55	84675	0.04171355	-0.23693974
LOC652433	652433	-0.421519182	-0.238542326
PARK2	5071	-0.405059788	-0.238542326
UBE2D2	7322	-0.688504674	-0.239345804
PHF21A	51317	-0.494408894	-0.240150847
TRIM69	140691	-0.39617818	-0.240150847
CXXC1	30827	-0.479244971	-0.242574817
BAZ1B	9031	-0.4645927	-0.244198295
RNF26	79102	-0.324982156	-0.245827941
ZNF278	23598	-0.412298125	-0.248283972
FBXO24	26261	-0.419659264	-0.251580308
PCGF5	84333	-0.517033567	-0.251580308
HERC6	55008	-0.06871578	-0.251580308
TRIM58	25893	-0.406858094	-0.254901854
RNF111	54778	-0.417807278	-0.254901854
MARCH9	92979	-0.28372514	-0.254901854
RNF180	285671	-0.207950137	-0.256572242
RNF149	284996	-0.253580978	-0.256572242
PJA2	9867	-0.262535809	-0.257409812
HERC5	51191	-0.322007463	-0.258249079
TRIM50A	135892	-0.227773746	-0.259089892
PHF2	5253	-0.460495466	-0.262469677
FBXO25	26260	-0.618613412	-0.262469677
RNF13	11342	-0.232572655	-0.26331871
TRAF4	9618	-0.332509036	-0.26502185

Gene Symbol	Entrez Gene ID	Log10 fold over Control (Replicate 1)	Log10 fold over Control (Replicate 2)
TRIM60	166655	-0.177151775	-0.26502185
TIF1	8805	-0.642747035	-0.269309033
RNF133	168433	-0.29611469	-0.270171636
LGR6	59352	-0.425263085	-0.279774523
RNF168	165918	-0.276991742	-0.279774523
RNF187	149603	-0.558426138	-0.280658104
PRICKLE1	144165	-0.332509036	-0.285103345
TRIM48	79097	-0.083011435	-0.285103345
UBE1L	7318	-0.592975553	-0.286894276
FBXO36	130888	-0.297513457	-0.286894276
WSB1	26118	-0.300324406	-0.288692539
RNF152	220441	-0.884159589	-0.289594471
SCEL	8796	-0.39617818	-0.290498364
SOCS3	9021	-0.429039661	-0.293221216
MNAT1	4331	0.064214566	-0.293221216
FBXL19	54620	-0.341717039	-0.295961247
TRIM37	4591	-0.159465533	-0.297797614
RNF190	162333	-0.111268185	-0.302422751
MKRN1	23608	-0.39093507	-0.306158602
WWP1	11059	0.09364928	-0.306158602
FBXO16	157574	-0.771841754	-0.307097586
DCUN1D3	123879	-0.131819252	-0.308038693
FBXL5	26234	-0.010000631	-0.310874134
RNF150	57484	-0.179280718	-0.31182338
KIAA0644	9865	-0.65533623	-0.31468372
FBXW8	26259	-0.658541338	-0.315641429
UBE2NL	389898	-0.415963155	-0.317563024
TRIM31	11074	-0.538455105	-0.323379632
PEX10	5192	-0.399709014	-0.327301014
UBE1L2	55236	-0.35912281	-0.330265486
RNF126	55658	-0.615688888	-0.332253134
FBXL17	64839	-0.343270852	-0.33525172
UBE2S	27338	-0.368919343	-0.336255836
UHRF2	115426	-0.089905128	-0.337262279
FBXO27	126433	-0.601354402	-0.341311727
FBXO3	26273	-0.519362207	-0.341311727
RNF166	115992	-0.4645927	-0.343350604
MLLT6	4302	-0.706089674	-0.346427171
TRIM17	51127	-0.417807278	-0.347457492
MARCH7	64844	-0.06214789	-0.35160349
BIRC3	330	-0.886328334	-0.358955736
ZNF364	27246	-0.438627029	-0.363213516
SH3MD4	344558	-0.512413274	-0.365358213
TRIM64	120146	-0.648996114	-0.365358213
BRAP	8315	-0.479244971	-0.365358213
CDC34	997	-0.479244971	-0.366434495
CISH	1154	-0.6782854	-0.368595197
UBR2	23304	-0.425263085	-0.368595197
ASB4	51666	-0.138567574	-0.374044405
TTC3	7267	-0.492209952	-0.378453583
HERC4	26091	-0.266430894	-0.391956023
PHF20L1	51105	-0.481379079	-0.393100371
BTRC	8945	-0.470811758	-0.395398261
UBE2N	7334	-0.76356931	-0.396551728
ZNF645	158506	-0.303153668	-0.396551728
LOC653111	653111	-0.490022087	-0.397708266
TNFRSF25	8718	-0.258675427	-0.398868001
FBXO33	254170	-0.743552149	-0.400030732
RCHY1	25898	-0.2823701	-0.401196694
RNF32	140545	-0.36074028	-0.403537831
C13ORF7	79596	-0.403268789	-0.40471326
FBXO41	150726	-0.213687281	-0.408258533
ASB12	142689	-0.307432511	-0.411832985

Gene Symbol	Entrez Gene ID	Log10 fold over Control (Replicate 1)	Log10 fold over Control (Replicate 2)
FLJ14627	84900	-0.553346664	-0.413030999
NDP52	10241	-0.935176758	-0.416645112
NEDD4L	23327	-0.237425109	-0.417856493
SMARCA3	6596	-0.826505754	-0.421511272
UBE2C	11065	-0.747482403	-0.422736322
FBXO39	162517	-0.548325912	-0.425196953
FBXL20	84961	-0.427147326	-0.430160322
TOPORS	10210	-0.234992105	-0.43518119
RKHD3	84206	-0.417807278	-0.43644547
ASB5	140458	-0.427147326	-0.437713442
ZNF330	27309	-0.713328233	-0.437713442
FBXW11	23291	-0.354306234	-0.438985245
MLLT10	8028	-0.540901816	-0.447993096
IRF2BP1	26145	-0.789309693	-0.45454369
UBE3C	9690	-0.205676361	-0.4558657
UBE2D4	51619	-0.187902212	-0.457191871
RNF175	285533	-0.349542394	-0.458521978
TRIM59	286827	-0.202287795	-0.459856298
TRAF3	7187	-0.648996114	-0.461194604
IBRDC1	154214	-0.313203893	-0.462537047
RNF14	9604	-0.716993151	-0.463883652
BAZ2B	29994	-0.269047108	-0.465234572
KUA-UEV	387522	-0.24604967	-0.466589581
LOC120824	120824	-0.107633881	-0.466589581
VPS18	57617	-0.906920281	-0.466589581
CCL20	6364	-0.618613412	-0.467948958
RNF135	84282	-0.186815127	-0.470680287
OIT3	170392	-0.639655989	-0.472052549
WDSUB1	151525	-0.392675714	-0.473429031
ZNF313	55905	-0.300324406	-0.476195284
RNF138	51444	0.183327651	-0.47897927
TRAF6	7189	-0.695453466	-0.480377918
C20ORF18	10616	-0.327977272	-0.481781086
RNF144	9781	-0.432849248	-0.484601227
LOC283116	283116	-0.36074028	-0.486018128
TRIM56	81844	-0.54336239	-0.488866009
TRIP	10293	-0.52642399	-0.488866009
MGRN1	23295	-0.590217977	-0.491732688
SMURF1	57154	-0.521703399	-0.494618279
MDM4	4194	0.04171355	-0.494618279
HECW1	23072	-0.642747035	-0.496068432
FBXO38	81545	-0.514717205	-0.49898321
DCUN1D4	23142	-0.385754609	-0.500447899
SH3RF2	153769	-0.652154603	-0.503392319
RNF130	55819	-0.45442138	-0.50487198
RFPL2	10739	-0.122357161	-0.510841837
LOC652859	652859	-0.372234551	-0.510841837
BRPF3	27154	-0.776037826	-0.512347142
MARCH6	10299	-0.645860239	-0.513857682
RNF103	7844	-0.423387099	-0.518421228
FBXL13	222235	0.224804638	-0.518421228
LL0XNC01-237H1.1	282808	-0.392675714	-0.519953081
ASB2	51676	-0.11401397	-0.5214905
FBXL3A	26224	-0.104029738	-0.5214905
BRCA1	672	-0.538455105	-0.523033237
ASB13	79754	-0.39617818	-0.524581618
WHSC1L1	54904	-0.582049269	-0.526135394
RFFL	117584	-0.958928417	-0.52925987
TRIM2	23321	0.129155072	-0.52925987
RFP2	10206	-0.923771112	-0.537170618
RNF122	79845	-0.17821491	-0.541987229
MAP3K1	4214	-0.136628775	-0.543604812
FBXO30	84085	-0.642747035	-0.546858013

Gene Symbol	Entrez Gene ID	Log10 fold over Control (Replicate 1)	Log10 fold over Control (Replicate 2)
TRIM36	55521	-0.448431075	-0.550135613
FBXO40	51725	-0.337088682	-0.553438292
PCGF6	84108	-0.759491438	-0.553438292
UBE2J1	51465	-0.373901796	-0.560119966
FBXL15	79176	-0.483523726	-0.561806493
ZNF294	26046	-0.444482834	-0.563499594
CHFR	55743	-0.521703399	-0.565199321
PCGF3	10336	-0.52642399	-0.566905887
UBE2H	7328	-1.254848979	-0.575540291
SOCS2	8835	-0.76356931	-0.577287899
CAND2	23066	-0.848857011	-0.577287899
CGRRF1	10668	-0.53360265	-0.580804519
BFAR	51283	-0.604183664	-0.582573482
TRAF7	84231	-0.472904726	-0.584349847
FBXL22	283807	-0.590217977	-0.587924188
ASB1	51665	-0.685071321	-0.589722619
FBXW9	84261	-0.406858094	-0.589722619
RNF165	494470	-0.512413274	-0.593341639
TRIM4	89122	-0.615688888	-0.593341639
ASB8	140461	0.00491522	-0.595162693
LOC51255	51255	-0.674931792	-0.598827524
ZNF216	7763	-0.129910282	-0.600671775
UBE2G1	7326	-0.607031478	-0.600671775
TRIM39	56658	-0.54336239	-0.604383592
RNF185	91445	-0.494408894	-0.611904129
AOF1	221656	-0.864595721	-0.613804701
IBRDC3	127544	-0.139540228	-0.615713805
MLL4	9757	-0.203414359	-0.615713805
ZNF650	130507	-0.494408894	-0.619557015
FBXL14	144699	-0.45442138	-0.621491631
FBXO32	114907	-0.538455105	-0.621491631
RNF8	9025	-0.490022087	-0.623434722
FBXO2	26232	-0.550829033	-0.623434722
HECTD3	79654	-0.910353424	-0.625386544
CHD5	26038	-0.706089674	-0.625386544
FBXO22	26263	-0.75144855	-0.627347363
DPF2	5977	-0.636586788	-0.63328313
LOC342897	342897	-0.868229968	-0.635279822
BIRC7	79444	-0.841931963	-0.635279822
SIAH2	6478	-0.444482834	-0.635279822
FBXO10	26267	-0.65533623	-0.639301149
RNF123	63891	-0.636586788	-0.639301149
SOCS1	8651	-0.563565884	-0.64336006
BIRC4	331	-0.627507036	-0.645403734
UBE2L3	7332	-1.291945473	-0.645403734
TRIM23	373	-0.771841754	-0.645403734
CBLL1	79872	-0.75144855	-0.649520355
LOC649055	649055	-0.919056925	-0.653676175
RNF146	81847	-0.685071321	-0.653676175
JARID1D	8284	-0.458461317	-0.653676175
LOC642446	642446	-0.512413274	-0.65576929
RAPSN	5913	-1.039138771	-0.65576929
SOCS5	9655	-1.035292066	-0.666388309
LRSAM1	90678	-0.789744635	-0.668543549
FBXO43	286151	-0.392675714	-0.672886588
KIAA1718	80853	-0.410477199	-0.675074401
RFP	5987	-0.477121298	-0.677273291
ZNF592	9640	-0.372234551	-0.681704964
CBLC	23624	-1.046149981	-0.68393777
NEDD4	4734	-0.450418677	-0.686182326
RNF141	50862	-0.706089674	-0.686182326
ASB16	92591	-0.496619027	-0.690706328
LOC652436	652436	-0.747482403	-0.690706328

Gene Symbol	Entrez Gene ID	Log10 fold over Control (Replicate 1)	Log10 fold over Control (Replicate 2)
PHF17	79960	-1.005686411	-0.690706328
TRIM43	129868	-0.354306234	-0.692986018
TRIM49	57093	-0.921407622	-0.697581831
PHF23	79142	-0.75144855	-0.704567937
VPS11	55823	-0.45442138	-0.709288417
HRC	3270	-0.598543453	-0.709288417
DTX3L	151636	-0.716993151	-0.716466983
TRIM10	10107	-0.483523726	-0.716466983
WDR24	84219	-0.417807278	-0.718886603
FBXO8	26269	-1.092518717	-0.72131955
ZA20D1	56957	-0.128958934	-0.723766203
C17ORF27	57674	-0.24729584	-0.726226949
RNF17	56163	-0.636586788	-0.731190435
FBXO34	55030	-0.731970156	-0.733693496
KIAA0317	9870	-0.340168767	-0.736211068
CBL	867	-0.872421301	-0.74385279
UBE2D3	7323	-0.281019274	-0.746430368
TRIM7	81786	-0.698970004	-0.746430368
MARCH2	51257	-0.850355426	-0.749023092
HIP2	3093	-0.621557763	-0.751631387
UBE2U	148581	-0.709693984	-0.751631387
HECTD2	143279	-0.652154603	-0.754255688
LPXN	9404	-1.08045422	-0.756895695
FBXL2	25827	0.03784744	-0.767619704
TRIM35	23087	-1.042240904	-0.773082416
MID1	4281	-0.724417095	-0.775840016
RING1	6015	-0.052478595	-0.778614977
ANAPC11	51529	-0.784553854	-0.778614977
LMO7	4008	-0.196698398	-0.778614977
RFPL1	5988	-0.100455258	-0.778614977
RNF10	9921	-0.519362207	-0.787048124
DTX4	23220	-0.521703399	-0.789895871
FBXW7	55294	-0.225393995	-0.789895871
MARCH3	115123	-0.946890427	-0.792762683
LMTK3	114783	-0.76356931	-0.792762683
PML	5371	-0.671603881	-0.792762683
PHF8	23133	-0.728177206	-0.795648275
RAB40A	142684	-0.6782854	-0.79855344
TRIM40	135644	-0.373901796	-0.79855344
LOC120824	120824	-0.405059788	-0.801477895
RNF6	6049	-1.057286382	-0.801477895
UBE2Z	65264	-0.75144855	-0.801477895
RNF167	26001	-0.392675714	-0.804422177
UBE2O	63893	-0.582049269	-0.804422177
ASB3	51130	-0.853871964	-0.810371587
SH3MD2	57630	-0.844392537	-0.813376996
FBXL8	55336	-0.75144855	-0.816403633
RNF121	55298	-0.674931792	-0.819451224
LOC652759	652759	-0.294720499	-0.822520352
VPS41	27072	-0.132776894	-0.828724745
PHF3	23469	-0.841441516	-0.831860648
UNKL	64718	-1.137597093	-0.838200613
LOC399940	399940	-0.485679016	-0.841405946
HECW2	57520	-0.790180014	-0.844634808
NLRC5	84166	-0.709693984	-0.854468288
PARC	23113	-1.265128674	-0.861149962
RNF20	56254	-0.604183664	-0.861149962
FBXO9	26268	-0.314658803	-0.871369105
NEURL2	140825	-0.627507036	-0.871369105
ZMYND11	10771	-0.563565884	-0.871369105
PJA1	64219	-1.022462267	-0.874829521
RNF24	11237	-0.731970156	-0.881834515
LOC646754	646754	-0.322007463	-0.885379676

Gene Symbol	Entrez Gene ID	Log10 fold over Control (Replicate 1)	Log10 fold over Control (Replicate 2)
ASB7	140460	-0.558426138	-0.885379676
ISL1	3670	-1.347966022	-0.888954352
CNOT4	4850	-0.674931792	-0.892558355
UBE2E1	7324	-0.798092402	-0.899857692
WSB2	55884	-0.759491438	-0.899857692
PHF21B	112885	-0.423387099	-0.911041835
UBE3B	89910	-0.297513457	-0.911041835
TRIM61	391712	-0.65533623	-0.93034707
RFPL4B	442247	-1.148393682	-0.934313125
ASB14	142686	-0.818528293	-0.938315733
UEVLD	55293	-0.845380684	-0.94643373
ASB11	140456	-1.421519182	-0.954706366
FBXL12	54850	-0.425263085	-0.967418305
ZNRF3	84133	-0.901820577	-0.967418305
UBE1C	9039	-0.481379079	-0.967418305
LOC92312	92312	-0.077912027	-0.971739534
CUL4A	8451	-0.444482834	-0.972173949
LOC440456	440456	-0.571391144	-0.983626956
RNF215	200312	-0.743552149	-0.993559203
TRIM67	440730	-0.83366848	-0.997228077
PXMP3	5828	-1.031479087	-1.011268831
FBXO21	23014	-0.971309617	-1.012221199
C1orf166	79594	0.248152836	-1.022349591
ITCH	83737	-0.545837137	-1.032719843
FBXL6	26233	-1.100455313	-1.043856167
CUL2	8453	-1.24729584	-1.08864623
LINCR	93082	-0.856908971	-1.089215085
UBE2T	29089	-1.160486192	-1.096678324
PHF12	57649	-0.79987046	-1.119259615
RNF170	81790	-0.627507036	-1.12109337
RNF25	64320	-0.728177206	-1.124784271
UBE2L6	9246	-0.865113041	-1.127884127
TRIM8	81603	-0.988845867	-1.134782815
SHFM3	6468	-0.555878975	-1.139869786
DTX1	1840	-0.460495466	-1.145664804
PHF13	148479	-1.47500783	-1.1469631
ANKIB1	54467	-1.324982156	-1.147613707
ZNF547	284306	-0.923179032	-1.15815863
LONRF2	164832	-1.178214975	-1.171710249
RBBP6	5930	-0.966706955	-1.18286451
MARCH1	55016	-1.023206552	-1.21434357
FLJ31951	153830	-1.109447204	-1.21434357
LONRF1	91694	-0.871369428	-1.238542326
TRIM11	81559	-1.120489186	-1.240150847
UBE2F	140739	-0.658541338	-1.285103345
MEFV	4210	-1.08045422	-1.291404058
DPF1	8193	-1.354306234	-1.312774705
UBE2V1	7335	-0.822733395	-1.330265486
HBXAP	51773	-0.590217977	-1.402365685
RNF2	6045	-1.421519182	-1.442822915
CBLB	868	-1.07875772	-1.478979139
PHF14	9678	-1.251056028	-1.486018128
LOC729974	729974	-1.276991742	-1.533989066
FLJ25076	134111	-1.15138536	-1.749023092
